# Traditional Medicinal Plants as a Source of Antituberculosis Drugs: A System Review

**DOI:** 10.1155/2021/9910365

**Published:** 2021-09-08

**Authors:** Yuhui Xu, Bowen Liang, Chengcheng Kong, Zhaogang Sun

**Affiliations:** ^1^Institute of Chinese Materia Medica, China Academy of Chinese Medical Science, Beijing 100700, China; ^2^Department of Traditional Chinese Medicine, Beijing Chest Hospital, Capital Medical University, Beijing 101149, China; ^3^Translational Medicine Center, Beijing Chest Hospital, Capital Medical University, Beijing 101149, China; ^4^Beijing Key Laboratory in Drug Resistant Tuberculosis Research, Beijing Tuberculosis & Thoracic Tumor Research Institute, Beijing 101149, China

## Abstract

Medicinal plants are the chief components in the different oriental formulations in different traditional medical systems worldwide. As a thriving source of medicine, the medicinal plants with antituberculosis (TB) properties inspire the pharmacists to develop new drugs based on their active components or semimetabolites. In the present review, the anti-TB medicinal plants were screened from the scientific literatures, based on the botanical classification and the anti-TB activity. The obtained anti-TB medicinal plants were categorized into three different categories, *viz.*, 159 plants critically examined with a total 335 isolated compounds, 131 plants with their crude extracts showing anti-TB activity, and 27 plants in literature with the prescribed formula by the traditional healers. Our systemic analysis on the medicinal plants can assist the discovery of novel and more efficacious anti-TB drugs.

## 1. Introduction

Globally, traditional medicines (TM) make a vital contribution to the health care industry. In some countries, TM is the main source of health care or even the sole health care service available, especially in the rural sector [1]. The popularity of TM is also increasing in the developed countries for many different reasons, one of which is that the effectiveness of these TM was proved by the ethnopharmacological research. Early in 1972, the World Health Organization (WHO) established a Department of Traditional Medicine (DTM). Later, WHO (2013) called on to strengthen its public services of the traditional medicine [1]. Recently, the International Classification of Traditional Medicine (ICTM) was added as a new chapter into the International Classification of Diseases—11 (ICD-11) [2]. This achievement currently refers to the Traditional Chinese Medicine (TCM) alone, which opens its doors to accommodate many other thriving traditional health care philosophies prevailing globally, such as Ayurveda and Traditional African medicine (TAM).

The spread of tuberculosis (TB) occurred from East Africa to the rest of the world with the migration of Homo sapiens, especially along the established trade routes with increased mingling and crowding of populations [3, 4]. Currently, there exist more than 10 million new cases of active disease and nearly 1.3 million deaths annually [5, 6]. In response to this spreading route, different countries developed their own traditional anti-TB formulations during the long courses in fighting this old plaque. Reports relating TB can be found in many ancestral data of the TM medical system, especially the TCM, Ayurveda, and TAM for its long history coexisting with human kinds for an estimated 70,000 years [7]. Investigations on the TM formulations show that the plants or herbs are the main composition of the traditional anti-TB formula, from which the active components or semimetabolites present a thriving source of new drugs. In the last 20 years, nearly 50% of drugs approved by the FDA in the United States of America have been derivatives of the natural products, including natural plant products [8]. Among the 435,000 plant species reported worldwide [9], an estimated 70,000 species of plants are used for medicinal purposes [10]. Thus, selecting plants based on ethnobotanical knowledge can enhance the probability to find new compounds with anti-TB activity.

Before this review, some articles summarized the role of local medical plants but only few with anti-TB purpose [11, 12]. In this review, the anti-TB medicinal plants in different countries or regions are included to analyze their botanical classification, active botanical parts, extract method, and *in vitro* anti-TB activities in brief. Subsequently, the effective anti-TB plants are described with the following three branches: those with the isolated effective compounds, those with their crude plant extracts showing anti-TB effect, and those only found in the formula prescribed by traditional healers. Finally, we discuss the influencing factors on the development of traditional medicine and its future trend. This review is to inspire the development of possible new anti-TB agents derived from plants.

## 2. Brief Description of the Overall Anti-TB Medicinal Plants

We present the data by searching the main three databases: Wangfang Med, Chinese National Knowledge Infrastructure, and PubMed. Combinations of the following search terms are used: “tuberculosis,” “plant,” “herb,” “Chinese and western medicine,” and “random.” In the present review, only the nonrepetitive plant species with good *in vivo* or *in vitro* anti-TB effect were accepted, although the criterion of the effectiveness was quite different with the inhibition concentration expressed in several different ways in different *Mycobacteria*, especially the *M. tuberculosis* H37Rv and the clinical isolates. The plants employed for treating the fever in traditional medicine have not been included, as fever is taken to be a nonspecific indication of many infections that are not restricted to TB. The exception to this is where fever is treated in conjunction with other TB-related symptoms like coughing.

**Figure 1:**
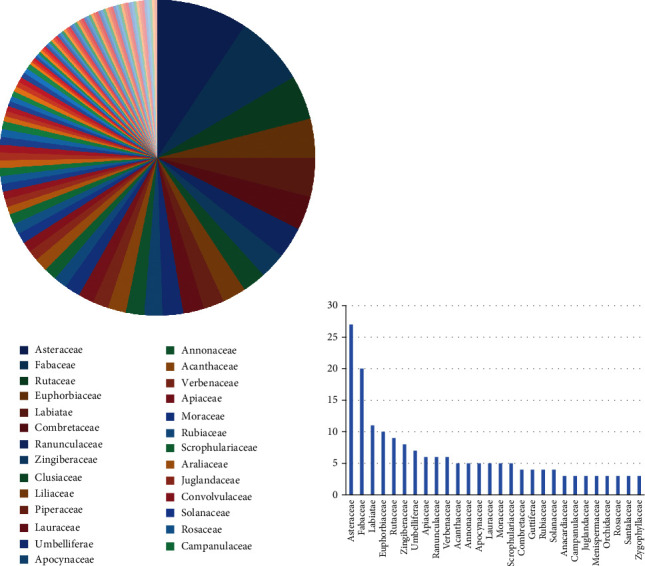
Classification of traditional anti-TB medicinal plants with effective crude extracts and the compounds. (a) Botanical families consisting of the anti-TB medicinal plants. There are 108 families including 230 genus and 277 species in this summary. (b) Genus number (>2) of the anti-TB medicinal plant families.

The classification of the traditional anti-TB medicinal plants in the present review belongs to 90 families including 230 genus and 277species (Figure 1). The top 11 families with more than 7 plant species include Fabaceae (21 species in 18 genus), Asteraceae (20 in 16 genus), Euphorbiaceae (14 in 11 genus), Lamiaceae (13 in 11 genus), Rutaceae (14 in 10 genus), Combretaceae (9 in 4 genus), Piperaceae (9 in 1 genus), Zingiberaceae (8 in 3 genus), Annonaceae (7 in 6 genus), and Apiaceae (7 in 7 genus). Forty plant families are only reported once. A total of 6 *Terminalia* genus that belongs to the Combretaceae family have up to 6 anti-TB plant species, and about 9 anti-TB plant species belong to only one genus *Piper*.

The literatures that we studied reported the anti-TB properties of the plant species from different plant parts (aerial parts, almonds, bark, bulbs, branches, fruits, flowers, heart woods, leaves, rhizomes, roots, stems, seeds, shoots, twigs, tubers, wood, whole plants, and even the ethnomedicinal recipes). With the leaves (83 cases), roots (61), aerial parts (32), barks (30), stems (14), whole plants (9), seeds (9), fruits (8), rhizomes (8), and flowers (7) are the top 10 most used anti-TB plant parts. For the same plant species, different parts of the plant presented a varied anti-TB effect. The useful plant parts of the genus *Lantana*, *Piper*, and *Terminalia* mainly focused on the leaves, leaves, and both leaves and roots, respectively.

It was observed that the extraction methods of the medicinal plants available in the literature significantly affected the anti-TB results. The general problems concerning the antibacterial screening of medicinal plant extracts have already been discussed in the literature [13]. There is still no single extraction method that is regarded as a standard for extracting the bioactive compounds from medicinal plants. One or more of the following solvents were mainly used in the studies: dichloromethane (268 times), methanol (65), ethanol (45), hexane (29), chloroform (18), ethyl acetate (11), water (11), and acetone (10), while diethylether, acetate, and hydroalcoholic solutions were seldom used. Since the extraction process deeply influences the results of the bioactivity tests and the subsequent isolation of bioactive compounds, selection of the best extraction method by consulting the traditional knowledge about the preparation of the herbal remedy remains crucial [14].

As noted earlier and evidenced by this review, many reports lack adequate statistical analysis of their results and appropriate controls for their anti-TB activity, while some studies lack the generic extraction schemes or tests against a panel of various species of *Mycobacteria* to avoid false positive results. In this review, the parallel cytotoxic evaluation on mammalian cell lines has not been provided, since our main aim was to summarize the crude extracts or compound precursors of the anti-TB medicinal plants, although this needs to be overcome in the future.

## 3. Compounds from the Plants with Anti-TB Activity

Different from the conventional process of drug discovery involving the screening of large molecular libraries for biological activities and/or in silico data mining approaches based on cheminformatics modeling, the bioactivity-guided fractionation was mostly employed in medicinal plants to isolate the bioactive compounds. They were extracted first from the specified parts of the plants, then fractionized and characterized by infrared spectroscopy, mass spectrometry, and nuclear magnetic resonance spectroscopy, to obtain the structural data. Finally, their bioactivities were verified in different mycobacteria.

Several groups summarized the active anti-TB natural products from the different organs and regions. Early in 2007, Copp and Pearce [15] summarized a total of 353 natural products (secondary metabolites) with reported growth inhibitory activity towards *M. tuberculosis* or related organisms from terrestrial and marine plants and animals and microorganisms. Abedinzadeh et al. [16] stressed the natural antimycobacterial peptides from bacteria, fungi, plants, and animals. Chinsembu [17] described the natural antimycobacterial agents from endophytes and medicinal plants in different regions of Africa, Europe, Asia, South America, and Canada. The present review only focused on the medicinal plants and the plants with anti-TB components belonged to the 156 species, 123 genus, and 64 families, of which Fabaceae (13 species, 10 genus), Rutaceae (10 species, 7 genus), and Lamiaceae (9 species, 7 genus) were the top three families; accordingly, more genera belong to those family with anti-TB activity (Table 1).

**Figure 2:**
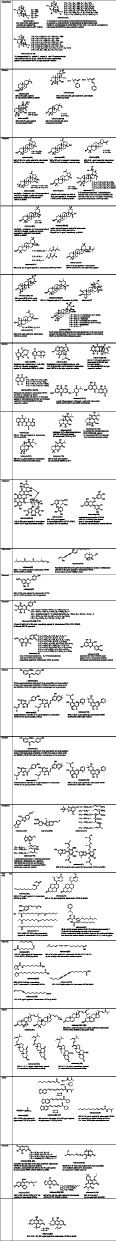
Typical structures sorted out of the 335 anti-TB compounds from the reported medicinal plants, which included sesquiterpene, diterpene, triterpene, quinone, polyphenol, fatty alcohol, flavanone, flavonoid, chalcone, benzofuran, fatty acid ester, fatty acid, steroid, amide, and coumarin.

Many plants consisted several components with anti-TB activity, and only the active compounds that were reported are listed in this review. Table 1 presents the list of 335 compounds, which were tested for their anti-TB activities. Those 335 compounds could be divided into mainly 11 classes, such as terpenes (37 types), ketones (31), acids (14), alcohols (10), esters (9), hydrocarbons (9), quinones (8), furans (7), phenols (6), and quinolones (3). The typical structures of the 335 compounds are sorted out in Figure 2. Of all the anti-TB natural compounds, the derivatives and analogs of phytol, flavones, and terpenoids were critically reviewed by Singh et al. [173] and Cantrell et al. [174] for their pharmacological activities of various diseases. These 335 compounds were natural products or secondary metabolites, and few of their synthetic modified derivatives have been mentioned in this review.

In fact, many semisynthetic derivatives proved to be more active than the parent compounds; for example, the methylation of natural compounds of mulinenic acid and 13-hydroxy-mulin-11-en-20-oic acid methyl ester decreased the minimum inhibitory concentration (MIC) by 8 times [42]; n-propyl ester and n-butyl ester of isomulinic acid decreased the MIC by 4 times [42]. The triacetylated methyl gallate decreased the MIC 2-4 times, since the acetylation increased the lipophilic nature of methyl gallate [19]. The abietane diterpenoid had an MIC of 1.2 *μ*g/ml, while its C-12 acetate analogue was more active with an MIC of 0.89 *μ*g/ml [175]. One of the most impressing natural products was (+)-calanolide A, a novel dipyranocoumarin from the Malesian tree *Calophyllum lanigerum* var. austrocoriaceum. This distinct compound was first reported with good activity against the strains of HIV-1, which was resistant to diverse other nonnucleosides as well as nucleoside (AZT) reverse transcriptase inhibitors [176, 177]. Later, the novel calanolides with the ring-D-modification were synthesized with selective activity against the replication and/or nonreplicating *M. tuberculosis* by targeting the Rv2466c [55]. In particular, analogues bearing 2-nitrofurano group at the ring D position markedly improved the *in vitro* efficacy and reduced the mammalian cell toxicity, when compared with the parent compound (+)-calanolide A [55]. Recently, Mu et al. demonstrated that the nitrofuranyl calanolides could be employed as novel fluorescent probes that can serve as a much needed high-throughput and low-cost detection method for detection of living *M. tuberculosis* and can precisely determine the MIC values for a full range of available drugs [178]. Thus, different modifications of the calanolide derivatives demonstrated three aspects (anti-HIV, anti-TB, and TB diagnosis) of potent usage in TB disease.

Of all the 335 natural plant compounds and its semisynthetic analogues, only few were found for their mechanistic role of their anti-TB activities. The calanolides target the Rv2466c, and hyperenone A inhibits the ATP-dependent MurE ligase, which involves in the cytoplasmic steps of peptidoglycan biosynthesis [104]. It was reported that saussureamine C (methyl 3-O-feruloylquinate) targets the folC [151] and eupractenoid B targets the acetyl transfer activity of GlmU [90]. The trans, trans-1, 7-diphenylhepta-4, 6-dien-3-one target the efflux pumps [26]. *In silico* analysis revealed that some fatty acids could bind at the cleft between the N-terminal and C-terminal lobes of the cytosolic domain of serine/threonine protein kinase B (PknB) [23]. The anti-TB plant medicinal compounds included in this review lack the molecular basis of the action and mechanisms of modulation on the metabolism of *M. tuberculosis* nor the immunomodulatory activities of those compounds.

## 4. Plants Showing Anti-TB Effect in Form of Crude Extracts

The plants whose active components were isolated and tested for their anti-TB activity as described in Section 3. This section summarizes the reported plants for their anti-TB activity only in the form of crude extracts. They are listed in Table 2 with the total amount of 128 plant species. The top five plant families were *Asteraceae*, *Euphorbiaceae*, *Fabaceae*, *Piperaceae*, and *Acanthaceae*, and the plant parts mainly used for extraction were root for *Fabaceae* family and leaf for *Asteraceae*, *Piperaceae*, and *Acanthaceae*, respectively. For *Euphorbiaceae* family, the plant parts of bark, fruit, leaf, root, and seed were reported with the anti-TB function.

Among the extraction methods, ethanol, hexane, and methanol were found to be the top three frequently used extracting solvents, while chloroform, dichloromethane, acetone, and ethyl acetate were used to a lesser extent. Of course, aqueous extract method was also popularly used, which involved the process of soaking, boiling, or/and hydrodistilling. Although the plant part used for study does not determine the extraction method, as a general rule, low molecule organic solvents are recommended when there is no reference.

To date, no specific cut-off value has been established as a reference to analyze the anti-TB activity results of the plant extracts, and many different methods are available to evaluate the activity. As of date, only a few anti-TB plant extracts in Table 2 have been tested in preclinical or clinical trials. It is encouraging that more and more promising crude extracts are now paving a way for the clinical test for their therapeutic applications. This section can provide a new perspective in expanding the anti-TB plant species for the development of anti-TB medicine in the future.

## 5. Medicinal Plants Only Found in Formula Prescribed by the Traditional Healers

Traditional healers continually serve the public health in most of the countries. Some ethnomedical information has been published based on many plant species in anti-TB formulas documented by different traditional healing systems, ranging from the poor documented oral African medicine to the well-documented Ayurveda or Chinese medicine. These reports inspired the scientists to find more effective compounds for tuberculosis. The investigations of medicinal plants using frontier technologies are now being reconsidered to be a feasible approach for discovering novel bioactive compounds and crude extracts, in order to solve the wide spreaded TB problems.  Table 3 shows the main species or families of the traditional plant medicines and their botanical details in the published papers by the systemic survey of the prescribed formula, but very few studies reveal the working compounds or active crude extracts.

The anti-TB formulas were investigated in many countries or regions, such as China, India, Mexico, South Africa, Pakistan, Iraq, Malaysia, Congo, Lao PDR, Nigeria, Nigeria, Burundi, Papua New Guinea, Lake Victoria Basin (Uganda, Kenya, and Tanzania), Arabian Peninsula, Southeast Asian, and Manus Province. Most anti-TB formulas were found during the ethnopharmacological investigation of the indigenous plants. To strengthen the anti-TB purpose, the present review summarized the anti-TB plant in the formula from the ethnopharmacological publications, with an emphasis on their classification (Table 3). Although three reports in Table 3 did not show the botanical family of the anti-TB medical plants in detail, it still can be speculated that the family *Fabaceae* is the most represented species, followed by *Asteraceae*, *Euphorbiaceae*, and *Liliaceae* families.

We need to be aware that the plant medicine used by the traditional healers is based on their according ethnomedical traditional medical theories. In comparison with the western system of medicine, the traditional plant medicines showed certain drawbacks. An important issue of all was that the active components of the herbal drugs prescribed were unknown. The activity of the traditional herbal drugs prescribed by the traditional healers can be greatly affected by the difference in the processing methods, variation in the potency due to difference in plant species and subspecies, varying geographical location of growth, nonuniform quality control standards, etc. Furthermore, for a given plant, a specific place, part, method, and time for collection can significantly affect the therapeutic efficacy [16, 40]. Hence, the plant medicine used by the traditional healers needs a critical evaluation to find the active components.

## 6. Need for Future Research

Although this review presents a big list of plants with effective anti-TB activities from different traditional medicine systems, there is a need of better therapeutic drug monitoring systems and high throughput *in vitro* assays for the routine screening to identify potentially serious and clinically significant herb-drug interactions [262]. Furthermore, there is a lack of *in vivo* information regarding the drug metabolism associated interactions with reference to the traditional medicines and the treatment of TB. This requires health care practitioners to ensure a clear communication with patients regarding the possible negative impacts of simultaneous use of certain TMs and prescribed drugs. It was reported that the widely used *Sutherlandia frutescens* in the treatment of TB in countries of Southern African Development Community interfered with the isoniazid therapy, but the mechanism of this interaction was not clear [263, 264]. Coadministration also resulted in the reduced bioavailability of ofloxacin [265], while piperine showed the ability to increase the bioavailability of the antituberculosis drug rifampicin [266–268].

Several issues affect the anti-TB activity of the components of the medicinal plants, such as the variation in the potency due to difference in species, absence of an integrated coding for every species used commonly in TMs, varying geographical location of growth and incorrect identification of drugs, and nonuniform quality control standards [269]. No clinic trial was reported on the crude extracts, and even the pure compounds from the medical plants still need to be elucidated for their constituent characterization and the mechanism of action. Till date, not many compounds have been originated from plants for further modification for use in clinic. We hope that this review will help to find a possible way to get better anti-TB results by making a combination of the compounds originated from plants based on the different TB-killing mechanisms.

## Figures and Tables

**Table 1:** Medicinal plants and natural products showing *in vitro* anti-TB activity.

Plant species	Plant family	Part used	Extracts	Compound class	Active constituents	References
Abrus precatorius	Fabaceae	Aerial parts	Dichloromethane fraction	Isoflavanquinone	Abruquinone B (1) against *M.* tuberculosis H37Ra with MIC of 12.5 *μ*g/ml by MABA^a,b^	[18]
Acacia farnesiana	Mimosaceae	Fruit	Methanolic extract	Parabens, flavanones	Methyl gallate (2) showed activity against the sensible strain *M.* tuberculosis H37Rv with MIC of 50 *μ*g/ml, respectively. The (2*S*)-Naringenin 7-*O*-*β*-galloylglucopyranoside (3) showed activity against multidrug resistant *M. t*uberculosi*s* G122 with MIC of 50 *μ*g/ml by MABA	[19]
Aglaia forbesii	Meliaceae	Leaf	Dichloromethane fraction	Benzopyran flavaglines	Desacetylpyramidaglain D (4) against *M*. tuberculosis Ra with MIC of 25 *μ*g/ml by MABA	[20]
Allanblackia floribunda	Guttiferae	Root bark	Successively macerated in dichloromethane-methanol (1 : 1) and methanol for 4 h	Biflavonoids	Morelloflavone (5) with the MIC of 19.53 and 39.06 *μ*g/ml against *M.* smegmatis and *M.* tuberculosis, respectively, by MABA	[21]
Allium neapolitanum	Alliaceae	Bulb	Chloroform extract	Canthinone	Canthin-6-one (6), 8-hydroxy-canthin-6-one (7), and 5(*ζ*)-hydroxy-octadeca-6(E)-8(Z)-dienioc acid (8) with MICs in the range 8–32 *μ*g/ml against a panel of fast-growing Mycobacterium species by dilution method	[22]
Allium sativum	Liliaceae	Bulb	Petroleum ether extract	Fatty acids	Lauric acid (9) and myristic acid (10) with MIC of 22.2 and 66.7 *μ*g/ml, respectively, against *M.* tuberculosis H37Ra by MABA	[23]
Allophylus edulis	Sapindaceae	Leaf	Hydrodistillation	Cycloprop[e]azulen-4-ol	Viridiflorol (11) against M. tuberculosis H37Rv (ATCC27294) with MIC of 190.0 *μ*g/ml by the microplate resazurin assay	[24]
Alnus incana	Betulaceae	Bark	Methanol extract	Triterpenes	Betulin (12), betulinic acid (13), and betulone (14) with MIC of 12.5, 84, and 57 *μ* g/ml against *M.* tuberculosis H37Ra by the microplate resazurin assay	[25]
Alpinia katsumadai	Zingiberaceae	Seed	n-Hexane	Diarylheptanoids	Trans,trans-1,7-diphenylhepta-4,6-dien-3-one (15) as efflux inhibitors against *M.* smegmatis mc^2^ 155 by thiazolyl blue tetrazolium bromide method	[26]
Amphipterygium adstringens	Anacardiaceae	Stem bark	Dichloromethane/methanol (1 : 1)	Tirucallanes	(14*β*, 24E)-3-oxolanosta-7,24-dien-26-oic acid (16) and (14*β*,24E)-3-hydroxylanosta-7,24-dien-26-oic acid (17) with MIC of 64 and 32 *μ*g/ml against *M*. tuberculosis H37Rv (ATCC 27294) by Bactec 460-TB apparatus	[27]
Amyris elemifera	Rutaceae	Leaf	Chloroform extract	Texalin	Texalin (18) with MIC of 25 *μ*g/ml against *M.* tuberculosis H37Rv by Bactec 460-TB radiometric methodology	[28]
Androsace umbellata	Primulaceae	Whole plants	Ethanol extract	Saxifragifolin	Saxifragifolin D (19) reduced the intracellular replication of *M.* tuberculosis in THP-1-derived macrophages but not in A549 cells	[29]
Angelica sinensis	Apiaceae	Root	Chloroform extract	Fatty diol	Falcarindiol (20), 9Z,17-octadecadiene-12,14-diyne-1,11,16-triol,1-acetate (21), and oplopandiol (22) with MIC of 26.7, 25.3, and 50.2 *μ*g/ml, respectively, against *M.* tuberculosis H37Rv by MABA	[30]
Anisochilus harmandii	Lamiaceae	Aerial parts	Water	Diterpenes	One pimarane-type diterpene named pimaric acid (23) and two abietane-type diterpenes named 9a,13a-epidioxyabiet-8 (14)-en-18-oic acid (24) and 15-hydroxydehydroabietic acid (25) with MIC of 50 *μ*g/ml, respectively, against *M*. tuberculosis H37Ra by MABA	[31]
Aralia nudicaulis	Araliaceae	Rhizome	Methanolic extract	Polyacetylene	(3R)-falcarinol (26) and (3R, 9R, 10S)-panaxydol (27) with MICs of 25.6 *μ*M and 36.0 *μ*M and IC50^f^s of 15.3 *μ*M and 23.5 *μ*M against *M.* tuberculosis H37Ra by microplate resazurin assay	[32]
Ardisia gigantifolia	Primulaceae	Leaves and stems	Chloroform extract	Resorcinol	5-alkylresorcinols (28), 5-(8Z-heptadecenyl) resorcinol (29), and 5-(8Z-pentadecenyl) resorcinol (30) with MIC of 34.4 and79.2 *μ*M against *M.* tuberculosis H37Rv (ATCC 27294) by MABA assay, respectively	[33]
Argyreia speciosa	Convolvulaceae	Root	Methanolic extract	Flavanoid sulphates	Quercetin 3′7 di-O methyl 3-sulphate (31) and kaempferol 7-O methyl 3-sulphate (32) against *M*. tuberculosis H37Rv with MIC values of 25 *μ*g/ml, respectively, by MABA	[34]
Arisaema sinii	Araceae	Whole plant	80% ethanol	Fatty acid ester	(E)-2-(methyl (phenyl) amino) ethyl 2-(2-hydroxyundecanamido)-7 (33), 11-dimethyl-3-oxotetradec-4-enoate (34), and compound 1 inhibit mycobacterial biofilm formation, disperse the preformed biofilms, and disrupt the mature biofilms at concentration of 4, 8, and 32 *μ*g/ml, respectively	[35]
Aristolochia brevipes	Aristolochiaceae	Rhizome	Dichloromethane extract	Benzo[f]-1,3-benzodioxolo[6,5,4-cd]indol-5(6H)-one	Aristolactam I (35) with MIC of 12.5-25 *μ*g/ml against drug resistant *M.* tuberculosis by fluorometrit MABA	[36]
Aristolochia taliscana	Aristolochiaceae	Hook roots	Hexanic extract	Neolignans	Licarin A (36), licarin B (37), and eupomatenoid-7 (38) with MIC of 3.12-12.5 *μ*g/ml against *M.* tuberculosis strains: H37Rv, four mono-resistant H37Rv variants and 12 clinical MDR isolates by MABA	[37, 38]
Arracacia tolucensis	Umbelliferae	Aerial parts	Dichloromethane–methanol (1 : 1)	Coumarins	Isoimperatorin (39), osthol (40), suberosin (41), and 8-methoxypsoralen (42) with MIC of 64, 32, 16, and 128 *μ*g/ml against *M.* tuberculosis H37Rv (ATCC 27294) by MABA	[39]
Artemisia capillaris	Asteraceae	Aerial parts	Methanol extracts	Stilbene derivatives	Ursolic acid (43) and hydroquinone (44) with MIC of 12.5-25 *μ*g/ml against *M.* tuberculosis MDR/XDR strains by MABA	[40]
Artocarpus lakoocha	Moraceae	Root	Dichloromethane extract	Benzofuran	Lakoochin A (45) and lakoochin B (46) with MIC of 12.5 and 50 *μ*g/ml against *M.* tuberculosis H37Ra by MABA	[41]
Azorella compacta	Umbelliferae	Aerial parts	n-Hexane	Diterpenoid	Azorellanol (47) and 17-acetoxy-13-alpha-hydroxy-azorellane (48) showed the strongest activity, with MIC of 12.5 *μ*g/ml against both strains (*M.* tuberculosis H37Rv (ATTC 27294) and a clinical isolate CIBIN/UMF15:99) by MABA	[42, 43]
Bauhinia purpurea	Fabaceae	Root	Dichloromethane extract	Dibenz[b,f]oxepin	Bauhinoxepin J (5,6-dihydro-3-methoxy-1,4-dionedibenz [b,f]oxepin (49) against *M.* tuberculosis H37Ra with a MIC of 24.4 *μ*M by MABA	[44]
Bauhinia saccocalyx	Fabaceae	Root	Dichloromethane extract	Dibenz[b,f]oxepin	Bauhinoxepins A (=3,3,5-trimethy lbenzo[b] pyrano[g] benzoxepin-6,11-diol) (50) and B (=6-methoxy-7-methyl-2-(3-methylbut-2-enyl)dibenzo[b,f]oxepine-1,8-diol (51) against *M.* tuberculosis H37Rv with MIC of 6.25 and 12.5 *μ*g/ml, respectively, by MABA	[45]
Beilschmiedia erythrophloia	Lauraceae	Root	Methanol	Endiandric acid	Erythrophloin C (52) and suberosol B (53) against *M.* tuberculosis H37Rv with MICs of 50 and 28.9 *μ*g/ml, respectively, by MABA	[46]
Beilschmiedia tsangii	Lauraceae	Leaf	Methanol	Epoxyfuranoid lignans	Three new epoxyfuranoid lignans, 4a,5a-epoxybeilschmin A (54) and B (55), and beilschmin D (56), together with known beilschmin A (57) and B (58) with MICs of 30, 40, 50, 2.5, and 7.5 *μ*g/ml, respectively, against *M.* tuberculosis 90-211378 by proportion method on agar	[47]
Blepharodon nitidum	Asclepiadaceae	Whole plant	Ethanol extract	Hydroperoxycycloartanes	24-Hydroperoxycycloart-25-en-3*β*-ol (59) and 25-hydroperoxycycloart-23-en-3*β*-ol (60) with MIC of 12.5 and 25 *μ*g/ml against drug-resistant clinical isolates by MABA	[48]
Bocconia arborea	Papaveraceae	Aerial parts	Chloroform extract	Dihydrochelirubine	Alkaloids 6-methoxydihydrochelirubine (61) and 6-methoxydihydrocheleritrine (62) against *M*. tuberculosis H37Rv and MDR tuberculosis with MIC of 12.5-50 *μ*g/ml by MABA	[49]
Caesalpinia pulcherrima	Fabaceae	Root	Dichloromethane	Cassane-furanoditerpenoids	6 Beta-benzoyl-7 beta-hydroxyvouacapen-5 alpha-ol (63) and 6 beta-cinnamoyl-7beta-hydroxyvouacapen-5 alpha-ol (64) with MIC of 25 and 6.25 *μ*g/ml against *M.* tuberculosis H37Ra by MABA	[50]
Caesalpinia sappan	Fabaceae	Heartwood	Methanol	Chalcone	3-Deoxysappanchalcone (65) against both drug-susceptible and drug-resistant strains of *M*. tuberculosis at MIC50s of 3.125–12.5 *μ*g/ml in culture broth and MIC50s of 6.25–12.5 *μ*g/ml inside macrophages and pneumocytes	[51]
Calliandra californica	Fabaceae	Root	Ethyl acetate	Cassane-type diterpenes	Escobarine A (66) and B (67) with MIC of 25-50 *μ*g/ml against *M.* tuberculosis H37Rv	[52]
Callicarpa pilosissima	Verbenaceae	Leaves and twigs	Methanol extract	Diterpenoids	12-Deoxy-11,12-dihydro-seco-hinokiol methyl ester (68), callicarpic acid B (69), and alpha-tocopherol trimer B (70) with MICs ≤63.6 *μ*M against *M.* tuberculosis H37Rv in vitro by MABA	[53]
Calophyllum lanigerum	Clusiaceae	All plant materials	Methanol	Coumarin	(+)-Calanolide A (71) against *M.* tuberculosis with H37Rv with MIC of 3.13 *μ*g/ml by the radiometric BACTEC	[54, 55]
Camchaya calcarea	Asteraceae	Whole plant	Dichloromethane	Cyclodeca[b]furan	Goyazensolide (72), centratherin (73), lychnophorolide B (74), isogoyazensolide (75), isocentratherin (76), 5-epi-isogoyazensolide (77), and 5-epi-isocentratherin (78) with MICs of 3.1, 3.1, 6.2, 1.5, 3.1, 3.1, and 3.1 *μ*g/ml, against *M.* tuberculosis H37Rv, respectively, by MABA	[56]
Celastrus vulcanicola	Celastraceae	Leaf	Dichloromethane extract	Sesquiterpenes	1*α*-Acetoxy-6*β*, 9*β*-dibenzoyloxy-dihydro-b-agarofuran (79) with MIC value of 6.2 *μ*g/ml against sensitive and resistant *M.* tuberculosis strains by MTT method	[57]
Chamaedorea tepejilote	Arecaceae	Leaf	Hexane extracts	Pentacyclic triterpenes, fatty alcohols	Ursolic acid (43) and farnesol (80) against *M.* tuberculosis H37Rv (ATCC 27294) with MIC of 50 *μ*g/ml and 8 *μ*g/ml, respectively, by MABA	[58, 59]
Chrysanthemum morifolium	Asteraceae	Flower	Methanol extract	3-Hydroxy triterpenoids	Maniladiol (81), 3-epilupeol (82), and 4,5a-epoxyhelianol (83) with MIC of 4 *μ*g/ml, 4 *μ*g/ml, and 6 *μ*g/ml, respectively, against *M.* tuberculosis H37Rv by MABA	[60]
Citrullus colocynthis	Cucurbitaceae	Fruit	Methanolic extract	Triterpenes	Ursolic acid (43) and cucurbitacin E 2-0-*β*-D-glucopyranoside (84) against *M.* tuberculosis H37Rv with MICs0f50 and 25 *μ*g/ml, respectively, by BACTEC 460TB system	[61]
Citrus aurantiifolia	Rutaceae	Fruit peels	Hexane extract	Furo[3,2-G]coumarin, fatty acid	Both 5,8-dimethoxypsoralen (85) and palmitic acid (86) with the MIC of 25 *μ*g/ml against *M*. tuberculosis H37Rv (ATCC 27294) and *M*. tuberculosis H10 by MABA	[62]
Clausena excavata	Rutaceae	Rhizome	Chloroform extract	Coumarins, carbazole derivatives	Dentatin (87), nor-dentatin (88), clausenidin (89), 3-formylcarbazole(90), mukonal (91), 3-methoxycarbonylcarbazole (92), 2-hydroxy-3-formyl-7-methoxycarbazole (93), and clauszoline J (94) with MIC of 50, 100, 200, 100, 200, 50, 100, and 100 *μ*g/ml against *M.* tuberculosis H37Ra by MABA	[63]
Clavija procera	Theophrastaceae	Stem and bark	Ethanol extract	Oleanane triterpene	Oleanane triterpenoid aegicerin (95) with MIC values ranged between 1.6 and 3.12 *μ*g/ml against 37 different sensitive and resistant MTB strains by thiazolyl blue tetrazolium bromide method	[64]
Clinacanthus siamensis	Acanthaceae	Leaf	Ethanol	Amide	Trans-3-methylthioacrylamide (96) with the MIC of 200 *μ*g/ml against *M*. tuberculosis H37Ra by MABA	[65]
Cnidoscolus chayamansa	Euphorbiaceae	Leaf	Chloroform : methanol (1 : 1)	Pentacyclic triterpenes	Moretenol (97) and moretenyl acetate (98) showed MIC of 25 *μ*g/ml against *M.* tuberculosis H37Rv and four monoresistant strains	[66]
Combretum molle	Combretaceae	Stem bark	Acetone	Polyphenol	Punicalagin (99) with MIC of 600 *μ*g/ml against *M.* tuberculosis H37Rv by the agar proportional method	[67, 68]
Cordia globifera	Ehretiaceae	Root	A hexane-soluble extract	Quinones	Globiferin (100) and cordiachrome C (101) with MICs of 6.2 and 1.5 *μ*g/ml, respectively, against *M*. tuberculosis H37Ra by MABA	[69]
Croton kongensis	Euphorbiaceae	Leaf	Dichloromethane	Diterpenedione	16-Dien-9,15-dione (102), ent-8,9-seco-8,14-epoxy-7R-hydroxy-11â-acetoxy-16-kauren-9,15-dione (103), ent-8,9-seco-7R-hydroxy-11â-acetoxykaura-8 (14) with MICs of 25.0, 6.25, and 6.25 *μ*g/ml, respectively, and possessed antimalarial activity with IC_50_ ranges of 1.0-2.8 *μ*g/ml against *M.* tuberculosis H37Ra by MABA	[70]
Curcuma longa	Zingiberaceae	Rhizomes	Chloroform extracts	Curcumin	Curcuminoid demethoxycurcumin (104) with MIC of 200 *μ*g/ml against *M.* tuberculosis H37Rv by BACTEC 460	[71, 72]
Curtisia dentata	Curtisiaceae	Leaf	Ethanol extracts	Triterpenes	Ursolic acid acetate (105) and betulinic acid acetate (106) with MIC of 3.4 *μ*g/ml and 19.8 *μ*g/ml against *M*. tuberculosis H37Rv (ATCC 27294) by MABA	[73]
Cynanchum atratum	Asclepiadaceae	Roots	Ethanol extract	Isoquinolin	(-)-Deoxypergularinine (107) with MIC of 12.5 *μ*g/ml against *M*. tuberculosis H37Ra, H37Rv, MDR, and XDR strains by Bactec MGIT 960TM	[74]
Derris indica	Fabaceae	Stem, root	Hexane : dichloromethane (1 : 1)	Flavonoids	3-Methoxy-(3^″^,4^″^-dihydro-3^″^,4^″^-diacetoxy)-2^″^,2^″^-dimethylpyrano-(7,8 : 5^″^,6^″^)-flavone (108), 3,4-methylenedioxy-10-methoxy-7-oxo[2]benzopyrano[4,3-b]benzopyran (109), karanjachromene (110), and pinnatin (111) against *M.* tuberculosis H37Ra with MICs of 25, 6.25, 12.5, and 12.5 *μ*g/ml by MABA	[75, 76]
Diospyros anisandra	Ebenaceae	Stem bark	n-Hexane extract	Naphthalene	Plumbagin (112) and 3,3′-biplumbagin(113) against *M.* tuberculosis H37Rv with MIC of 1.56 and 3.33 *μ*g/ml by MABA	[7]
Diospyros decandra	Ebenaceae	Bark	Hexane	Triterpenes	2-Oxo-3b,19a-dihydroxy-24-nor-urs-12-en-28-oic acid (114) with MIC of *μ*g/ml against *M.* tuberculosis H37Ra by MABA	[77]
Diospyros montana	Ebenaceae	Stem bark	Chloroform extract	Quinonoids	Plumbagin (112) > emodin (115) > menadione (116) > thymoquinone (117) > diospyrin (118) against *M*. tuberculosis H37Ra (ATCC 25177) and MDR-TB by MABA	[78, 79]
Disthemonanthus benthamianus	Fabaceae	Stem bark	Methanol extract	Flavonoids	Distemonanthoside (119), sitosterol 3-O- -d-glucopyranoside, 4-methoxygallic acid (118), and quercetin (121), against a clinical isolate strain of *M.* tuberculosis AC 45 with MIC ranged from 31.25 to 125 *μ*g/ml by MABA	[80]
Dracaena angustifolia	Dracaenaceae	Leaf	Dichloromethane extract	Triterpenes, fatty acids, fatty alcohols	Ergosterol-5,8-endoperoxide (122), linoleic acid (123), and E-phytol (124) with MICs ≤ 2 *μ*g/ml against *M.* tuberculosis H37Rv (ATCC 27294) by MABA	[81]
Ehretia longiflora	Boraginaceae	Root	Methanolic extract	Quinone	Ehretiquinone (125) and prenylhydroquinone (126) with MIC of 25 and 26.2 *μ*g/ml against *M.* tuberculosis strain H37Rv by the agar proportion method	[82]
Engelhardia roxburghiana	Juglandaceae	Root	Methanol	Quinone	Engelharquinone (127), 3-methoxyjuglone (128), and (-)-4-hydroxy-1-tetralone (129) with MIC of 3.125, 3.125, and 6.25 *μ*g/ml against M. tuberculosis 90-221387 and 0.2, 0.2, and 4.0 *μ*g/ml against *M*. tuberculosis H37Rv, respectively, by agar proportion method	[83, 84]
Eriosema chinense	Fabaceae	Root	Exane and dichloromethane extracts	Flavonoids	Khonklonginol A (130), B (131), H (132), and lupinifolinol (133), dehydrolupinifolinol (134), flemichin (135), eriosemaone A (136), and lupinifolin (137), with MICs of 25, 50, 25, 25, 12.5, 12.5, 12.5, and 12.5 *μ*g/ml, respectively, against *M*. tuberculosis H37Ra by MABA	[85]
Erythrina gibbosa	Fabaceae	Ethnomedicinal recipes	70% aqueous methanol	Isoflavonoids	Isoflavonoidspasellidin (138) and erythobissin (139) with MICs, respectively, lying between 8 *μ*g/ml and 25 *μ*g/ml against MTB; 3-phenyl coumarin derivative indicanine (140) with MIC of 18.5 *μ*g/ml against *M.* smegmatis by agar proportion method	[86]
Erythrina indica	Fabaceae	Root bark	Successively extracted with dichloromethane–methanol (1 : 1) and methanol	Isoflavonoids	Indicanine B (141) with MIC of 18.5 *μ*g/ml against *M.* smegmatis by agar proportion method	[86]
Erythrina senegalensis	Fabaceae	Ethnomedicinal recipes	70% aqueous methanol	Isoflavonoids	Isoflavonoidspasellidin (138) and erythobissin (139) with MICs between 8 *μ*g/ml and 25 *μ*g/ml against *M.* tuberculosis H37Rv; 3-phenyl coumarin derivative indicanine (138) with MIC of 18.5 *μ*g/ml against *M.* smegmatis by agar proportion method	[86]
Eucalyptus torelliana	Myrtaceae	Leaf	Hexane extract	Fatty acid ester	Hydroxymyristic acid methylester (142) and a substituted pyrenyl ester (143), a sterol with MIC of 49.45 and 46.99 *μ*g/ml against *M.* tuberculosis H37Rv (ATCC 27294) by MABA	[87]
Euclea natalensis	Ebenaceae	Root	Acetone extract	Naphthoquinone	Diospyrin (118) and 7-methyljuglone (144) against *M.* tuberculosis H37Rv (ATCC 27294) by BACTEC 460 with four- to sixfold reduction of MIC	[88]
Euphorbia ebracteolata	Euphorbiaceae	Roots	80% ethanol	Diterpenoids	Rosane-type diterpenoids 3 (145) and 8 (146) displayed moderate inhibitory effects on with the MIC of 18 *μ*g/ml and 25 *μ*g/ml, respectively, by MABA	[89, 90]
Euphorbia lagascae	Euphorbiaceae	Air-dried powdered plant	Methanol extract	Steroids	Ergosterol peroxide (147), cycloart-23-en-3*β*,25-diol (148), vanillin (149), and 4-hydroxybenzaldehyde (150) against *M.* tuberculosis H37Rv ATCC 27294 strain using two different systems: BACTEC 460TB (Bactec 460)	[91]
Exocarpos latifolius	Santalaceae	Stem	Methanol extract	Fatty acid	Exocarpic acid (E-octadeca-13-ene-9,11-diynoic-acid) (151) with MIC of 20 *μ*g/ml against *M.* tuberculosis H37Ra (ATCC 25177) by the thiazolyl blue tetrazolium bromide method	[92]
Fatoua pilosa	Moraceae	Whole plant	Methanol extract	Coumarin; chalcones	Scopoletin (152), isobavachalcone (153) and (E)-1-[2,4-dihydroxy-3-(3-methylbut-2-enyl)phenyl]-3-(2,2-dimethyl-8-hydroxy-2H-benzopyran-6-yl)prop-2-en-1-one (154), copoletin (155), and umbelliferone (156) with MICs of 42, 18, 30, 42, and 58.3 *μ*g/ml against *M.* tuberculosis H37Rv by the agar proportion method	[93]
Ferula hermonis	Apiaceae	Root	Ethanol extraction	Octahydroazulen	Jaeschkeanadiol benzoate (teferidin) (157) and jaeschkeanadiol p-hydroxybenzoate (ferutinin) (158) with MIC values of 3.125 and 1.56 *μ*g/ml against *M.* bovis BCG Pasteur 1173P2 and 0.69 and 2 *μ*g/ml against *M.* tuberculosis H37Rv, respectively, by fluorescence assay	[94]
Garcinia livingstonei	Guttiferae	Leaf	Acetone extract	Flavonoids	Amentoflavone (159) and 4-monomethoxy amentoflavone (160) with MIC of 0.6 ad 1.4 mg/ml against *M.* smegmatis (ATCC 1441), with the positive control isoniazid (MIC = 1.70 mg/ml) by tetrazolium violet indicator	[95]
Garcinia multiflora	Clusiaceae	Heartwood	Methanol	Biflavones	6,6^″^-Biapigenin hexamethylether (161), volkensiflavone hexamethylether (162), and hexamethylether of GB-1a (163) against *M.* tuberculosis H37Rv with inhibition of 96%, 95%, and 87% at 1.25 *μ*g/ml by BACTEC 460TB	[96]
Garcinia nobilis	Clusiaceae	Stem bark	Methanol	Flavonoids	4-Prenyl-2-(3,7-dimethyl-2,6-octadienyl)-1,3,5,8-tetrahydroxyxanthone (164) with MIC of 8 *μ*g/ml against *M.* tuberculosis H37Rv ATCC 27294 and *M.* tuberculosis clinical MTCS2 by MABA	[97]
Goniothalamus laoticus	Annonaceae	Flower	Ethyl acetate extract	Lactone derivative	(+)-Altholactone (165) and howiinin A (166) with MIC of 6.25 *μ*g/ml, respectively, against *M.* tuberculosis H37Ra by MABA	[98]
Harrisonia perforata	Simaroubaceae	Branches	Ethanolic extract	Flavonoids	Perforamone B (5-hydroxy-7-methoxy-2-methyl-8-(1-hydroxy-3-methyl-3-butenyl)chromone) (167) and D (2-hydroxymethylalloptaeroxylin) (168), peucin-7-methyl ether (169), and greveichromenol (170) with MICs of 25, 25, 50, and 50 *μ*g/ml against *M.* tuberculosis H37Ra by MABA	[99]
Helichrysum melanacme	Asteraceae	Shoots	Acetonic extract	Chalcone	2,′4′,6′-Trihydroxy-3′-prenylchalcone (171) and 4′,6′,5^″^-trihydroxy-6^″^,6^″^-dimethyldihydropyrano-[2^″^,3^″^-2′,3′] chalcone (172) with MICs of 0.05 *μ*g/ml against *M.* tuberculosis H37Rv by BACTEC radiometric method	[100]
Heracleum maximum	Apiaceae	Root	Methanolic extract	Furanocoumarins	(3R,8S)-Falcarindiol (173) and 6-isopentenyloxyisobergapten (174) with MICs of 24 *μ*M and 167 *μ*M and IC_50_s of 6 *μ*M and 27 *μ*M against *M.* tuberculosis H37Ra, respectively	[101]
Humulus lupulus	Cannabaceae	Strobile hops	Hexane extract	Fatty acid	Unsaturated fat oleic and linoleic acids (175) with MIC of 4 and 16 *μ*g/ml against *M.* fortuitum by thiazolyl blue tetrazolium bromide method	[102]
Hydnocarpus anthelmintica	Flacourtiaceae	Seeds	95% ethanol	Fatty acid	Anthelminthicins A (176), B (177), C (178) (11-(cyclopent-1-en-1-yl)-11-oxoundecanoic acid, 2,3-dihydroxypropyl 9-[(R)-cyclopent-2-en-1-yl]nonanoate, 2,3-dihydroxypropyl 13-[(R)-cyclopent-2-en-1-yl]tridecanoate), and two known ones, namely, chaulmoogric acid (179) and ethyl chaulmoograte (180) with MIC of 5.54, 16.70, 4.38, 9.82, and 16.80 *μ*M, respectively, by GFP-expressed *M.* tuberculosis H37Rv	[103]
Hypericum perforatum	Guttiferae	Aerial parts	Hexane and chloroform extracts	Pyranone	Hyperenone A (181) against *M.* tuberculosis H37Rv and *M.* bovis BCG with MIC of 75 *μ*g/ml and 100 *μ*g/ml, respectively, by thiazolyl blue tetrazolium bromide method	[104]
Indigofera longeracemosa	Fabaceae	Stem	Methanol extract	Diterpene	Diterpene 12-methyl-5-dehydroacetylhorminone (182) with MIC of 0.38 *μ*g/ml against *M.* tuberculosis H37Rv by proportion method	[105]
Ipomoea leptophylla	Convolvulaceae	Leaf	Organic soluble extract	Triterpenes	3â,25-Epoxy-3R,21R-dihydroxy-22â-angeloyloxyolean-12-ene-28-oic acid (183), camaric acid (184), and rehmannic acid (185) with MICs of 64, 64, and 32 *μ*M, respectively, against *M.* tuberculosis H37Rv by BACTEC 460 radiometric system	[106]
Juniperus communis	Cupressaceae	Needles and branches	Methanolic extracts	Diterpene	Isocupressic acid (186) and communic acid (187) displayed MICs of 78 *μ*M and 31 *μ*M against *M.* tuberculosis H37Ra, respectively, by microplate resazurin assay	[107]
Juniperus procera	Cupressaceae	Leaf and bark	95% ethanol	Diterpene	Plumbagin (112) and 7*β*-hydroxyabieta-8,13-dien-11,12-dione (188) with MIC < 12.5 *μ*g/ml against *M.* tuberculosis H37Rv by visible growth	[108]
Kaempferia galanga	Zingiberaceae	Rhizomes	Absolute ethanol	Aromatic acid	Ethyl p-methoxycinnamate (189) inhibited the growth of *M*. tuberculosis H37Ra and H37Rv with MICs of 48.5 and 24.2 mM by Bactec 460 system	[109]
Kaempferia parviflora	Zingiberaceae	Rhizomes	Water	Flavonoids	3,5,7,4′-Tetramethoxyflavone (190) and 5,7,4′-trimethoxyflavone (191) with MIC of 200 and 50 *μ*g/ml, respectively, against *M.* tuberculosis H37Rv by MABA	[110]
Lantana hispida	Verbenaceae	Aerial parts	Hexane extract	Pentacyclic triterpenoids	Three pentacyclic triterpenoids of 3-acetoxy-22-(2′-methyl-2Z-butenyloxy)-12-oleanen-28-oic acid (192), 3-hydroxy-22*β*-(2′-methyl-2Z-butenoyloxy)-12-oleanen-28-oic acid (reduced lantadene A) (193), and oleanolic acid (194) with MIC of 50, 50, and 25 *μ*g/ml against *M.* tuberculosis H37Rv by MABA	[111]
Lantana horrida	Verbenaceae	Aerial parts	Hexanic extracts	Triterpenes	Ursolic acid (43) and oleanolic acid (194) against *M*. tuberculosis H37Rv by MABA	[58]
Larrea divaricata	Zygophyllaceae	Aerial parts	Dichloromethane/methanol (1 : 1)	Flavone, polyphenols	5,7-Dihydroxy-3-methoxy-2-(4-methox-yphenyl)-4H-chromen-4-one (195); 5,6,7-trihy-droxy-3-methoxy-2-(4-methoxyphenyl)-H-chromen-4-one (196); *β*,*γ*-dimethyl-,*α*,*δ*-bis(3,4-dihydroxyphenyl)butane (17) with MIC of 50 *μ*g/ml, respectively, against *M*. tuberculosis H37Rv (ATCC 27294) by Bactec 460-TB apparatus	[27]
Ligusticum officinale	Apiaceae	Root	n-Hexane extract	Fatty alcohol	Falcarindiol (198) with MIC of 20 *μ*g/ml and sesquiterpene alcohol a-prethapsenol (199) with MIC of 60 *μ*g/ml against *M.* tuberculosis H37Rv by spot culture growth inhibition assay	[112]
Limnophila geoffrayi	Scrophulariaceae	Aerial parts	Chloroform extract	Flavonoids	Lavonesnevadensin (5,7-dihydroxy-6,8,4′-trimethoxyflavone) (200) and isothymusin (6,7-dimethoxy-5,8,4′-trihydroxyflavone) (201) against *M.* tuberculosis H37Ra by MABA	[113]
Lippia turbinata	Verbenaceae	Aerial parts	Methanol-dichlormethane	Triterpenoids	3*β*,25-Epoxy-3*α*,21*α*-dihydroxy-22*β*-(3-methylbut-2-en-1-oyloxy) olean-12-ene-28-oic acid (202); 3*β*,25-epoxy-3*α*,21*α*-dihydroxy-22*β*-angeloyloxyolean-12-ene-28-oic acid (203); 3*β*,25-epoxy-3*α*,21*α*-dihydroxy-22*β*-tigloyloxyolean-12-ene-28-oic acid (204); and 3*β*,25-epoxy-3R-hydroxy-22*β*-(2-methylbutan-1-oyloxy)olean-12-ene-28-oic acid (205), lantanilic acid (206), camaric acid (184), lantanolic acid (207), and rehmannic acid (185) against *M.* tuberculosis H37Rv (ATCC 27294) using the radiorespirometric BACTEC 460 system	[106]
Litsea hypophaea	Lauraceae	Root	Methanol	Lactone, phenol	Litseakolide L (208) and N-trans-feruloylmethoxytyramine (209) with MIC values of 25 and 1.6 *μ*g/ml, respectively, against M. tuberculosis H37Rv by agar proportion method	[76]
Lophira lanceolata	Ochnaceae	Roots	Methanol extract	Flavonoids	Dihydrolophirone A (210) and lophirone A (211) with MIC of 31.25 and 15.75 *μ*g/ml against *M.* tuberculosis by MABA	[114]
Lunasia amara	Rutaceae	Leaf	Ethanol	Phenyl quinoline	4-Methoxy-2-phenylquinoline (212) and 4-methoxy-2-(3,4-ethylenedioxy) phenyl quinoline (213) with MICs of 16 *μ*g/ml, respectively, against *M*. tuberculosis H37Rv by BACTEC radiometric method	[115]
Marsypopetalum modestum	Annonaceae	Stem	Ethanol	Dithiopyridine	Dipyrithione (214) with MIC < 0.039 *μ*g/ml, respectively, against *M*. tuberculosis H37Rv by MABA	[116]
Micromelum hirsutum	Rutaceae	Stem bark	Dichloromethane extract	Carbazole	Micromolide ((−)-*Z*-9-octadecene-4-olide) (215) and five known alkaloids: lansine (216), 3-formylcarbazole (90), and 3-formyl-6-methoxycarbazole (217) with MIC of 1.5, 31.5, 14.3, 42.3, and 15.6 *μ*g/ml, against *M*. tuberculosis H37Rv by MABA	[117]
Microtropis fokienensis	Celastraceae	Root	Methanol	Sesquiterpenes	1*α*-Acetoxy-2*α*-hydroxy-6*β*,9*β*,15-tribenzoyloxy-*β*-dihydroagarofuran (218), 2*α*-acetoxy-1*α*-hydroxy-6*β*,9*β*, 15-tribenzoyloxy-*β*-dihydroagarofuran (219), rbiculin G (220), and triptogelin G-2 (221) with MICs of 19.5, 15.8, 14.6, and 26 *μ*M against *M*. tuberculosis 90-221387 by agar proportion method	[118]
Microtropis japonica	Celastraceae	Stem	Methanol	Sesquiterpenes	15-Acetoxyorbiculin G (222), celahin C (223), salasol A (224), and 8-acetoxymutangin (225) with a MIC of 39.6 *μ*M, 15 *μ*M, 15 *μ*M, and 10 *μ*g/ml, respectively, against *M*. tuberculosis H37Rv by agar proportion method	[119, 120]
Morinda citrifolia	Rubiaceae	Leaves	Hexane fraction	Steroid	E-Phytol (mixture of the two ketosteroids, stigmasta-4-en-3-one (226) and stigmasta-4-22-dien-3-one (227)) and the epidioxysterol campesta-5,7,22-trien-3*β*-ol (228) with MIC of 2.5 *μ*g/ml and minus 2.0 *μ*g/ml against *M*. tuberculosis H37Rv (ATCC 27294) by the growth index	[121, 122]
Mulinum crassifolium	Apiaceae	Aerial parts	n-Hexane	Diterpenoid	13-Hydroxy-mulin-11-en-20-oic-acid methyl ester (229), isomulinic acid n-propyl ester (230), and isomulinic acid n-butyl ester (231) with MIC of 6.25 *μ*g/ml, respectively, against *M.* tuberculosis H37Rv (ATCC 27294) by MABA	[43]
Myrtus communis	Myrtaceae	Leaf	Hydrodistillation	Monoterpene	Essential oil of limonene (232), 1-8 cineole (233), and *α*-pinene (234) agains*t M*. tuberculosis H37Rv with MIC of 2% (*v*/*v*)	[123]
Nigella sativa	Ranunculaceae	Seed	Methanolic extract	Quinones	Thymoquinone (TQ; 2-isopropyl-5-methyl-1, 4-benzoquinone) (117) with MIC of 12.5 *μ*g/ml intracellular *M*. tuberculosis H37Rv and extensively drug-resistant tuberculosis (XDR-TB) 72 h postinfection in RAW 264.7 cells	[124]
Ocimum basilicum	Lamiaceae	Aerial parts (leaves, fruits, and flowers)	Methanolic extract	Fatty acid ester	(E)-3′-Hydroxy-4′-(1^″^-hydroxyethyl)-phenyl-4-methoxycinnamate (235) against *M*. tuberculosis H37Rv (ATCC 27294) by MABA	[125]
Ocimum sanctum	Lamiaceae	Leaf	Ethanol extract	Triterpenes	Ursolic acid (UA, 3b-hydroxy-urs-12-en-28-oic-acid) (43) with MIC of 12.5 *μ*g/ml against *M.* tuberculosis by MABA	[40, 126]
Ocotea macrophylla	Lauraceae	Wood, leaf	Ethanol extract	Apomorphine	(S)-3-Methoxynordomesticine hydrochloride (236) with MIC of 4 *μ*g/ml against *M.* tuberculosis H37Rv by thiazolyl blue tetrazolium bromide method	[127]
Oplopanax horridus	Araliaceae	Inner stem bark	Consecutively extracted with dichloromethane, methanol, and 50% aqueous methanol	Sesquiterpenes	Oplopandiol (237) and falcarindiol (198) with MI of 61.5 and 6.2 *μ*g/ml against *M.* tuberculosis (ATCC 35801) by MABA	[128]
Pedilanthus tithymaloides	Euphorbiaceae	Root	Successively using hexane, dichloromethane, and methanol	Diterpenoid	Caniojane (238) against *M.* tuberculosis H37Ra with MIC of 25 *μ*g/ml by MABA	[129]
Phoradendron robinsonii	Santalaceae	Whole plant	Dichloromethane/methanol (1 : 1)	Flavanone	5-Hydroxy-2-(4′-hydroxyphenyl)-7-methoxy-2,3-dihydro-4H-chromen-4-one(239) with MIC of 128 *μ*g/ml against *M.* tuberculosis H37Rv (ATCC 27294) by Bactec 460-TB apparatus	[27]
Physalis angulata	Solanaceae	Aerial parts	Chloroform	Physalin	Physalin D (240) with MIC of 32 *μ*g/ml against *M.* tuberculosis H37Rv ATCC 27294 by MABA	[130]
Piper chaba	Piperaceae	Root	95% ethanol	Amide	Pellitorine (241), guineensine (242), sarmentine (243), brachyamide B (244), 1-piperettyl pyrrolidine (245), sarmentosine (246), and 1-(3,4-methylenedioxyphenyl)-1E-tetradecene (247) with MIC values in the range of 25–200 *μ*g/ml against *M*. tuberculosis H37Ra by MABA	[99, 131]
Piper regnellii	Piperaceae	Leaves and stems	Supercritical fluid extraction (CO2 extracts)	Benzofuran	Eupomatenoid-3 (248) and 5-(3-methyl-5-propylbenzofuran-2-yl) benzo[d][1,3]dioxole (249) against *M*. tuberculosis H37Rv by colorimetric resazurin microtiter assay plate	[132]
Piper solmsianum	Piperaceae	Leaf	Methanolic extract	Benzofuran	Synergism was observed in *M*. tuberculosis H37Rv and eight clinical isolates with eupomatenoid-5 (250) + rifampicin and in *M.* tuberculosis H37Rv and 17 clinical isolates with eupomatenoid-5+ ethambutol combinations	[133]
Plectranthus grandidentatus	Lamiaceae	Aerial parts	Chloroform	Royleanone	Abietanes 6*β*,7*α*-dihydroxyroyleanone (251), horminone, and 6,7-dehydroroyleanone (252) with MIC of 25 and 12.5 *μ*g/ml, respectively, against *M.* tuberculosis H37Rv by MTT	[134]
Plectranthus ornatus	Lamiaceae	Aerial parts	Chloroform	Royleanone	7*α*-Acetoxy-6*β*-hydroxyroyleanone (MIC 0.0083 *μ*M) (253) and 6,7-dehydroroyleanone (MIC 0.039 *μ*M) (254) against *M.* tuberculosis H37Rv by thiazolyl blue tetrazolium bromide method	[134, 135]
Plumbago indica	Plumbaginaceae	Root	Petroleum ether	Naphthalene	Plumbagin (112) with MIC of 0.25 and 8 *μ*g/ml against MDR-TB and 2 and 4 *μ*g/ml against the XDR-TB isolates by thiazolyl blue tetrazolium bromide method	[78]
Plumeria bicolor	Apocynaceae	Leaves	Chloroform extract	Naphthalene	Plumericin (112) against active and MDR TB with MIC of 0.12, 0.15, 0.07, 0.13, and 0.14 *μ*g/ml, respectively, better than isoplumericin	[136]
Polyalthia cerasoides	Annonaceae	Root	Extracted successively with hexane, ethyl acetate, and methanol	Apomorphine, fatty acid, sesquiterpenes	Bidebiline E (255), octadeca-9,11,13-triynoic acid (256), and *α*-humulene (257), with 6.25 *μ*g/ml against *M*. tuberculosis H37Ra MABA	[137]
Polyalthia debilis	Annonaceae	Root	Methanol	Lactone derivative	Debilisones B (258), C (259), and E (260) with MIC of 25, 12.5, and 25 *μ*g/ml, respectively, against *M*. tuberculosis H37Ra by MABA	[138]
Polyalthia evecta	Annonaceae	Root	Extracted successively with hexane, dichloromethane, and methanol	Furan	Furanoid polyacetylene (261) with MIC of 3.1 *μ*g/ml against *M.* tuberculosis H37Ra by MABA	[139]
Potamogeton malaianus	Potamogetonaceae	Whole plant	Dichloromethane	Diterpenes	Potamogetonyde (262), potamogetonol (263), potamogetonin (264), and 15,16-epoxy-12-oxo-8 (17),13 (16),14-labdatrien-20,19-olide (265) with MIC of 50–100 *μ*g/ml against *M*. tuberculosis H37Ra by MABA	[140]
Pourthiaea lucida	Rosaceae	Leaf	Methanol	Alcohol	a-Tocospiro A (266) and B (267), a-tocopherylquinone (268), and (E)-phytol (124) with MICs of 30, 50, 25, and 12.5 *μ*g/ml against *M*. tuberculosis H37Rv by agar proportion method	[141]
Premna odorata	Lamiaceae	Leaf	Methanol	Aldehydes	1-Heneicosyl formate (269) with MIC of 8 *μ*g/ml against *M.* tuberculosis H37Rv (ATCC27294) by MABA	[142]
Prunus cerasoides	Rosaceae	Root	Successively with hexane, ethyl acetate, and methanol	Fatty acid	Octadeca-9,11,13-triynoic acid (256) with MIC of 6.25 *μ*g/ml against *M.* tuberculosis H37Ra by MABA	[137]
Punica granatum	Punicaceae	Peel of the fruit	Water	Polyphenol	Epigallocatechin-3-gallate (EGCG) (270) and quercetin (271) with MIC of MIC 32-256 *μ*g/ml against nine *M*. tuberculosis isolates by thiazolyl blue tetrazolium bromide method	[143]
Radermachera boniana	Bignoniaceae	Leaves and twigs	Ethyl acetate	*Sterol*	Ergosterol peroxide (147) and b-sitostenone (272) with MICs of 34.8 and 9.9 *μ*M, respectively, against *M.* tuberculosis H37Rv by MABA	[144]
Ranunculus ternatus	Ranunculaceae	Roots	Ethanol	Benzophenones	(benzophenones) Methyl (*R*)-3-[2-(3,4-dihydroxybenzoyl)-4,5-dihydroxyphenyl]-2-hydroxypropanoate (273) with MIC of 41.67 *μ*g/ml against *M.* tuberculosis H37Rv by MABA	[145]
Rumex hymenosepalus	Polygonaceae	Root	Dichloromethane/methanol (1 : 1)	Diphenylethylene	5-[(E)-2-(4-Acetoxyphenyl) ethenyl]-1,3-benzenediol(1a) (274) with MIC of 32 *μ*g/ml against *M*. tuberculosis H37Rv (ATCC 27294) by Bactec 460-TB apparatus	[27]
Rumex nepalensis	Polygonaceae	Root	Ethanol extracts	Glycoside	Rumexneposide A (275), torachrysone (276), nepodin-8-O-b-D-glucopyranoside (277), torachrysone-8-O-b-D-glucopyranoside (278), and chrysophanol-8-O-b-D-glucopyranoside (279), which showed MICs of 20.7, 6.1, 26.6, 8.9, and 4.1 *μ*M, respectively, by fluorescence assay	[146]
Salvia africanalutea	Lamiaceae	Aerial parts	Ethanol extract	Diterpene	Abietane-type diterpene carnosic acid (280) with MIC of 28 *μ*M against *M.* tuberculosis H37Rv (ATCC27294) by a rapid radiometric method	[147]
Salvia miltiorrhiza	Lamiaceae	Roots	Acetone	Tanshinones	Tanshinone I (281), tanshinone IIA (282), and cryptotanshinone (283) with MIC in the range of 1.17–26.57 *μ*g/ml against *M.* tuberculosis H37Rv by agar proportion method	[148, 149]
Sapium indicum	Euphorbiaceae	Fruit	Hexane extract	Phorbol ester	Sapintoxin A (284), sapintoxin B (285), 12-(2¢-N-methylaminobenzoyl)-4R-deoxy-5,20-dihydroxyphorbol-13-acetate (286), and 12-(2-methylaminobenzoyl)-4-deoxyphorbaldehyde-13-acetae (287) with MIC of 3.12, 12.5, 25, and 25 *μ*g/ml, respectively, against *M.* tuberculosis H37Ra by MABA	[150]
Saussurea lappa	Asteraceae	Bark	Ethanol extract	Sesquiterpenoids	Saussureamine C (methyl 3-O-feruloylquinate) (288) against *M.* tuberculosis by inhibiting folC	[151]
Scleropyrum wallichianum	Santalaceae	Twig	Successively with n-hexane, chloroform, and methanol	Fatty acid	Scleropyric acid (289) with MIC of 25 *μ*g/ml against *M.* tuberculosis H37Ra by MABA	[152]
Solanum torvum	Solanaceae	Fruit	Methanol extracts	Xanthine	Methyl caffeate (290) with MIC of 8 *μ*g/ml against *M.* tuberculosis by agar proportion method	[153]
Stephania dinklagei	Menispermaceae	Aerial parts	Chloroform extract	Flavanone	Flavanone pinostrobin (291) against *M.* tuberculosis H37Rv with MIC of 3.125 *μ*g/ml by MABA	[49]
Strobilanthes cusia	Acanthaceae	Leaf	Methanol extracts	Quinazoline	Tryptanthrin (292) with MIC of 1 mg/ml against *M.* tuberculosis by BACTECH	[154, 155]
Tabernaemontana citrifolia	Apocynaceae	Leaf	Chloroform extract	Voacangine	Ibogaine (293) and voacangine (294) with MIC of 50 *μ*g/ml against *M*. tuberculosis H37Rv by Bactec 460-TB radiometric methodology	[28]
Teloxys graveolens	Chenopodiaceae	Aerial parts	Acetone extract	Flavanone	Flavanone pinostrobin (291) against *M.* tuberculosis H37Rv with MIC of 12.5 *μ*g/ml by MABA	[49]
Terminalia avicennioides	Combretaceae	Root bark	Successively with petroleum ether, ethyl acetate, chloroform, and methanol	Triterpenes	Arjunolic acid (295) and friedelin (296) which, respectively, had MICs against BCG of 156 *μ*g/ml and 4.9 *μ*g/ml, respectively, by broth microdilution method	[156, 157]
Terminalia brownii	Combretaceae	Root	Methanol extract	Flavones, ellagic acid	Methyl (S)-flavogallonate (297), ellagic acid xyloside (298), and methyl ellagic acid xyloside (299) against *M.* smegmatis by measured spectrophotometrically at 620 nm	[158]
Byrsonima fagifolia	Malpighiaceae	Leaf	Chloroform extract	Amyrin	*α*- and *β*-Amyrin (300), *α*-amyrin acetate (301), and dotriacontane (302) with MIC of 31.25, 62.5, and 62.5 *μ*g/ml, respectively, against *M.* tuberculosis H37Rv (ATCC 27294) by MABA	[159]
Terminalia laxiflora	Combretaceae	Root	Methanol extract	Triterpenes, fatty alcohol, sterol	Friedelin (296), triacontanol (303), *β*-sitosterol (34), and sitostenone (35) with MIC of 250 *μ*g/ml, 250 *μ*g/ml, 500 *μ*g/ml, and 500 *μ*g/ml, respectively, against *M.* smegmatis by measured spectrophotometrically at 620 nm	[158]
Terminalia superba	Combretaceae	Stem bark	Methanol extract	Ellagic acid	3,4′-Di-O-methylellagic acid 3′-O-*β*-D-xylopyranoside (306) and 4′-O-galloy-3,3′-di-O-methylellagic acid 4-O-*β*-Dxylopyranoside (307) with MIC of 4.88 *μ*g/ml and 9.76 *μ*g/ml, respectively, against *M. tuberculosis* H37Rv (ATCC 27294) by MABA	[160]
Tetracera potatoria	Dilleniaceae	Stem bark	Methanol/dichloromethane (1 : 1)	Alcohol	Tetraceranoate (308) and N-hydroxy imidate-tetracerane (309) with MIC of 7.8 *μ*g/ml and 15 *μ*g/ml, respectively, against *M.* smegmatis (ATCC 23246) by tetrazolium method	[161]
Tetradenia riparia	Lamiaceae	Leaf	Hydrodistillation	Royleanone	6,7-Dehydroroyleanone (254) with MIC of 31.2 *μ*g/ml against M. tuberculosis H37Rv by resazurin microtiter assay	[162]
Thalia multiflora	Marantaceae	Aerial parts	Dichloromethane–methanol (1 : 1) followed by a 100% methanol	Steroids	Stigmast-5-en-3*β*-ol-7-one (310), stigmast-4-ene-6*β*-ol-3-one (311), stigmast-5,22-dien-3*β*-ol-7-one (312), and stigmast-4,22-dien-6*β*-ol-3-one (313) were found to be the most active compounds with MIC of 1.9, 4., 1.0, and 2.2 *μ*g/ml, respectively, against *M.* tuberculosis by fluorescence assay	[163]
Tiliacora triandra	Menispermaceae	Root	Dichloromethane	Bisbenzylisoquinoline alkaloids	Tiliacorinine (314), 2′-nortiliacorinine (315), and tiliacorine (316) are bisbenzylisoquinoline alkaloids with MIC of 3.1–6.2 mg/ml against *M.* tuberculosis different strains by MABA	[164]
Tussilago farfara	Asteraceae	Aerial parts	Soxhlet extracted	Aromatic acid	p-Coumaric acid (317) and 4-hydroxybenzoicacid (318) with MIC 31.3 *μ*g/ml of 62.5 *μ*g/ml against *M.* tuberculosis H37Rv by high throughput spot culture growth inhibition assay	[165]
Ventilago madraspatana	Rhamnaceae	Stem bark	Methanol	Anthraquinone	Emodin (115) with MIC of 4 *μ*g/ml against *M.* tuberculosis H37Rv by MABA	[78, 166]
Zanthoxylum capense	Rutaceae	Leaf	80% ethanol	Phenol, amide	Decarine (319) and an N-isobutylamide, N-isobutyl-(2E,4E)-2,4-tetradecadienamide) (320) with MICof 1.6 *μ*g/ml against *M*. tuberculosis H37Rv by measuring the optical density at 600 nm in a Tecan M200 plate spectrophotometer	[167]
Zanthoxylum leprieurii	Rutaceae	Stem bark	Methanol extract	Dihydroacridine	Hydroxy-1, 3-dimethoxy-10-methyl-9-acridone (321), 3-hydroxy-1, 5, 6--trimethoxy-9-acridone (322) with MIC of 5.1 and 1.5 *μ*g/ml, respectively, against *M.* tuberculosis H37Rv by MABA	[168]
Zanthoxylum schinifolium	Rutaceae	Leaf	Methanolic extracts	Coumarin	7-[(2E) −3,7-dimethylocta-2,6-dienoxy] −8-methoxychromen-2-one (collinin) (323) with MIC50 of 3.13–6.25 *μ*g/ml against both drug-susceptible and -resistant strains of *M.* tuberculosis by luminescent viability assay kit	[169]
Zanthoxylum wutaiense	Rutaceae	Root	Methanol extract	Benzofuran, furo[3,2-b]quinoline	7-Methoxyanodendroate (324), 7-methoxywutaifuranal (325), wutaiensal (326), dictamnine (327), and *γ*-fagarine (328), with MIC of 35, 35, 30, 30, and 30 *μ*g/ml, respectively, against *M. Tuberculosis* H37Rv by the agar proportion method	[170]
Zingiber cassumunar	Zingiberaceae	Root	Methanol	Three fatty acid esters	(E)-4-(3,4-Dimethoxyphenyl)but-3-en-1-yl linoleate (329), (E)-4-(3,4-dimethoxyphenyl)but-3-en-1-yl oleate (330), and (E)-4-(3,4-dimethoxyphenyl) but-3-en-1-yl palmitate (331),with MIC of 200, 100, and 200 *μ*g/ml, respectively, against *M.* tuberculosis H37Ra by MABA	[171]
Ziziphus cambodiana	Rhamnaceae	Root bark	Acetate extract	Triterpenes	3-O-Vanillylceanothic acid (332), betulinaldehyde (333), betulinic acid (334), and 2-O-E-p-coumaryl alphitolic acid (335) with MIC of 25, 25, 25, and 12.5 *μ*g/ml, respectively, against *M*. tuberculosis H37Ra by MABA	[172]

MABA: microplate alamar blue assay; MIC: minimum inhibitory concentration.

**Table 2:** Medicinal plants and their crude extracts showing *in vitro* anti-TB activity.

Plant species	Plant family	Part used	Extracts	References
Acacia catechu	Liliaceae	Root	Hexane extracts with inhibition of mycobacterial (standard and clinical) growth	[179]
Acacia senegal	Fabaceae	Root	Aqueous extraction has potential antimycobacterial activity	[180]
Acalypha indica	Euphorbiaceae	Leaf	Aqueous extracts with inhibition of 95% at 4 percent *v*/*v* concentration in L-J medium for sensitive *M.* tuberculosis H37Rv	[181]
Achyrocline alata	Asteraceae	Leaf, stem	Aqueous extracts against *M.* tuberculosis H37Rv strain (ATCC 27294) with MIC^a^ of 62.5 *μ*g/ml	[182]
Acorus calamus	Acoraceae	Root	Aqueous extracts against *M.* bovis BCG by OD units	[183]
Adhatoda vasica	Acanthaceae	Leaf	Methanol extracts with the oils inhibiting the growth of MTB B19-4 at 2 *μ*g/ml	[180]
Allium sativum	Liliaceae	Leaf	Aqueous extract was found to be 63% at 4 percent *v*/*v* concentration in L-J medium for sensitive *M*. tuberculosis H37Rv	[181]
Aloe vera	Xanthorrhoeaceae	Leaf	Aqueous extract was found to be 41% at 4 percent *v*/*v* concentration in L-J medium for sensitive *M.* tuberculosis H37Rv	[181, 184]
Alstonia scholaris	Apocynaceae	Leaf	Methanol extracts have potential antimycobacterial activity and the synergistic group consisting of rifampicin in murine model	[185]
Amborella trichopoda	Amborellaceae	Fruit	Methanol extracts against *M*. bovis BCG (strain 11-73 P2) with MIC of 2.5 *μ*g/ml	[186]
Ambrosia ambrosioides	Asteraceae	Aerial parts	Methanolic extracts against *M*. tuberculosis H37Rv with MIC of 790 *μ*g/ml	[187]
Ambrosia confertiflora	Asteraceae	Aerial parts	Methanol, chloroform, dichloromethane, and ethyl acetate extracts against *M.* tuberculosis H37Rv with MIC of 200, 90, 120, and 160 *μ*g/ml, respectively	[187]
Amphipterygium simplicifolium	Julianaceae	Leaf	Dichloromethane-methanol extracts (1.1) inhibit the *M*. tuberculosis H37Rv at 50 *μ*g/ml with 90.5 ± 1.0%	[188]
Andrographis paniculata	Acanthaceae	Aerial parts	Hexane and methanol (1 : 5) extracts with maximum antimycobacterial activity at 250 *μ*g/ml against all the tested strains of *M*. tuberculosis (H37Rv, MDR, and drug sensitive)	[189]
Andrographis paniculata	Acanthaceae	Leaf	Ethanol extracts with inhibition of mycobacterial (standard and clinical) growth	[7, 190]
Angiopteris evecta	Marattiaceae	Leaf	80% methanol extract against *M*. tuberculosis H37Rv ATCC 25618 with an MIC of 400 *μ*g/ml	[191]
Apodytes dimidiata	Icacinaceae	Leaf	Hexane extractions against the field strain of MDR-TB and against the *M*. tuberculosis H37Rv with MIC of 0.47 and 0.31 mg/ml, respectively	[192]
Artemisia ludoviciana	Asteraceae	Bark, leaf	Hexane extracts against MDR-TB clinical isolates with MIC of 25-100 *μ*g/ml	[58, 193]
Artemisia nilagirica	Asteraceae	Leaf	Ethanol extracts against *M*. smegmatis with IC50^c^ of 300 *μ*g/ml	[194]
Beilschmiedia obscura	Lauraceae	Root	Ethyl acetate extracts against *M.* tuberculosis H37Rv with MIC of 31.25 *μ*g/ml by MABA^b^	[195]
Bidens odorata	Asteraceae	Aerial parts	Hexane, dichloromethane, ethyl acetate, and ethanolic extracts against *M.* tuberculosis H37Rv (ATCC 27294) with MIC of 100, 12.5, 12.5, and 12.5 *μ*g/ml	[196]
Bridelia micrantha	Euphorbiaceae	Bark	Acetone extracts against *M.* tuberculosis H37Ra with MIC of 25 *μ*g/ml	[197]
Calluna vulgaris	Ericaceae	Aerial parts	Ethyl acetate extracts with 97% inhibition at 100 *μ*g/ml against *M*. tuberculosis H37Rv (ATCC 27294)	[198]
Calophyllum brasiliense	Clusiaceae	Leaf	Dichloromethane-methanol extracts (1.1) inhibit the *M.* tuberculosis H37Rv at 50 *μ*g/ml with 82.8 ± 0.4%	[188]
Capparis zeylanica	Capparidaceae	Leaf	Ethyl acetate extracts against *M*. tuberculosis H37Rv with the 32 mm minimum zone of inhibition	[199, 200]
Carya illinoensis	Juglandaceae	Bark	Hexane extracts against *M.* tuberculosis H37Rv with MIC of 31 *μ*g/ml	[193]
Cassia sophera	Caesalpiniaceae	Seed	Methanol extracts against *M.* smegmatis with MIC of 125 *μ*g/ml	[201]
Chenopodium ambrosioides	Amaranthaceae	Leaf	80% ethanol crude extracts against *M*. tuberculosis H37Ra (ATCC 25177™) with MIC of 5000 *μ*g/ml	[184]
Chrysactinia mexicana	Asteraceae	Root	Ethyl ether extract against a drug-resistant strain of *M*. tuberculosis CIBIN/UMF15:99 with MIC of 62.5 *μ*g/ml	[202]
Citrullus colocynthis	Cucurbitaceae	Leaf	Chloroform extracts against *M*. tuberculosis H37Rv with MIC of 2.5 mg/ml by MABA	[203]
Citrus sinensis	Rutaceae	Fruit peel	Hexane extracts against two drug-resistant strains of *M*. tuberculosis with MIC of 25 and 50 *μ*g/ml	[204]
Cladonia arbuscula	Cladoniaceae	Root	Hexane and ethyl acetate extracts with 96% and 99% inhibition at 100 *μ*g/ml against *M.* tuberculosis H37Rv (ATCC 27294), respectively	[198]
Cocculus hirsutus	Menispermaceae	Leaf	Ethanol extracts against *M.* tuberculosis H37Rv (ATCC 27294) with MIC of 500 *μ*g/ml	[196]
Codiaeum peltatum	Euphorbiaceae	Stem	Methanol extracts against *M*. bovis BCG (strain 11-73 P2) with MIC of 100 *μ*g/ml	[186]
Combretum aculeatum	Combretaceae	Aerial part	Aqueous extracts inhibiting *M.* marinum with MIC of 0.2 mg/ml	[205]
Costus speciosus	Zingiberaceae	Stem, flower	Hexane partition from methanol extracts against *M.* tuberculosis H37Rv with MIC of 100 *μ*g/ml	[206]
Cremaspora triflora	Rubiaceae	Leaf	Acetone extracts decreased 16-fold of MIC in combination with fifampicin against *M.* aurum and reduction of the MICs of the anti-TB drug ranged from 2-fold to 4-fold, 2-fold to 64-fold, and 2-fold to 64-fold for *M*. smegmatis, *M*. aurium, and *M*. tuberculosis, respectively	[207]
Croton sylvaticus	Euphorbiaceae	Leaf, root, stem bark	Decoction, not known	[208]
Curcuma caesia	Zingiberaceae	Rhizome	Ethanol extract against *M.* tuberculosis H37Rv (ATCC 27294) with MIC of 31.25 *μ*g/ml	[196]
Cymbopogon citratus	Poaceae	Stem, rhizome	Hexane partition from methanol extracts of 200 *μ*g/ml against *M*. tuberculosis H37Rv	[206]
Cyperus rotundus	Cyperaceae	Root	Ethanol extracts against *M*. tuberculosis H37Rv (ATCC 27294) with MIC of 62.5 *μ*g/ml	[209]
Davilla elliptica	Dilleniaceae	Leaf	Chloroform extracts showed a promising antimycobacterial activity with a MIC of 62.5 *μ*g/ml by MABA	[209]
Dissotis rotundifolia	Melastomataceae	Leaf	80% ethanol crude extracts against *M.* tuberculosis H37Ra (ATCC 25177™) with MIC of 5000 *μ*g/ml	[184]
Dryopteris stewartii	Dryopteridaceae	Whole plant	Decoction, not known	[203]
Echinops giganteus	Asteraceae	Root	Methanol extracts against *M.* tuberculosis H37Ra and H37Rv with MIC of 32 and 16 *μ*g/ml, respectively	[210]
Empetrum nigrum	Empetraceae	Root	Hexane extracts with 95% inhibition at 100 *μ*g/ml against *M*. tuberculosis H37Rv (ATCC 27294)	[198]
Erythrina abyssinica	Fabaceae	Root bark	Methanol extracts showed the highest activity on *M*. tuberculosis H37Rv (MIC 390 *μ*g/ml)	[211]
Eulophia nuda	Orchidaceae	Tubers	Ethanol extracts against *M*. tuberculosis H37Rv (ATCC 27294) with MIC of 500 *μ*g/ml	[196]
Euphorbia albomarginata	Euphorbiaceae	Shoots	Extracts by n-hexane, dichloromethane, ethyl acetate, and methanol individually against *M.* tuberculosis H37Rv with MIC of 250-1000 *μ*g/ml	[212]
Euphorbia hirta	Euphorbiaceae	Leaf	Ethyl acetate extracts showed better activity with maximum of 64.73% reduction in relative light units against *M*. tuberculosis H37R	[213]
Evodia elleryana	Rutaceae	Bark	Ethyl acetate extracts with 95% inhibition of *M*. tuberculosis H37Ra grown in vitro (ATCC 25177) at 50 *μ*g/ml	[214]
Ficus sur	Moraceae	Root	80% ethanol against *M.* tuberculosis H37Ra (ATCC 25177) with MICof 0.78 mg/ml	[215]
Ficus citrifolia	Moraceae	Leaf	95% ethanol extracts against *M*. tuberculosis H37Rv (ATCC 27294) with 91% inhibition at 100 *μ*g/ml	[216]
Flourensia cernua	Asteraceae	Leaf	Hexane extracts against sensitive and resistant strains, respectively, with MIC of 25-50 *μ*g/ml	[217]
Foeniculum vulgare	Umbelliferae	Aerial parts	Hexane extracts against *M*. tuberculosis H37Rv with MIC of 100 *μ*g/ml	[204]
Globularia alypum	Globulariaceae	Leaf	Petroleum ether extracts against *M*. tuberculosis H37Rv with IC_50_ of 77 *μ*g/ml	[218]
Glycyrrhiza glabra	Fabaceae	Root	Ethanol extracts against *M*. tuberculosis H37Rv (ATCC 27294) with MIC of 250 *μ*g/ml	[219]
Guaiacum coulteri	Zygophyllaceae	Flower	Methanol extracts against *M*. tuberculosis H37Rv with MIC of 1000 *μ*g/ml	[188]
Guiera senegalensis	Combretaceae	Aerial parts	Aqueous extracts inhibiting *M*. marinum with MIC of 200 *μ*g/ml	[205]
Gymnosperma glutinosum	Asteraceae	Leaf	Hexane extracts against *M*. tuberculosis H37Ra and H37Rv both at 31.2 *μ*g/ml	[220]
Helianthus annuus	Asteraceae	Stem	Extracts by n-hexane, dichloromethane, ethyl acetate, and methanol individually against *M.* tuberculosis H37Rv with MIC of 250-500 *μ*g/ml	[212]
Heracleum maximum	Apiaceae	Root	Aqueous extracts against *M.* bovis BCG by OD units	[183]
Heteromorpha trifoliata	Apiaceae	Leaf	Ethanol extracts against *M*. tuberculosis H37Rv with MIC of 80 *μ*g/ml	[221]
Hygrophila auriculata	Acanthaceae	Root, leaf	Acetone extract against *M*. tuberculosis H37Rv by y the disc diffusion method	[222]
Juglans mollis	Juglandaceae	Bark	Hexane extracts against *M.* tuberculosis H37Rv with MIC of 50 *μ*g/ml	[193]
Juglans regia	Juglandaceae	Bark, leaf	Hexane extracts against *M*. tuberculosis strain H37Rv with MIC of 100 *μ*g/ml	[193, 194]
Justicia adhatoda	Acanthaceae	Leaf	Ethanolic extract against *M.* tuberculosis H37Rv by y the disc diffusion method	[223]
Khaya senegalensis	Meliaceae	Bark, leaf	Ethanolic extracts against *M.* tuberculosis H37Ra with MIC of 6.25 *μ*g/ml	[224]
Lantana camara	Verbenaceae	Leaf	Methanol extracts against *M*. tuberculosis H37Rv with MIC of 20 *μ*g/ml	[17]
Lantana hispida	Verbenaceae	Leaf	Hexane extracts against drug-resistant clinical isolates of *M.* tuberculosis with MIC of 100-200 *μ*g/ml	[193, 194]
Laurelia novae-zelandiae	Monimiaceae	Leaf, flower	Aqueous extract against *M*. smegmatis with IC_50_ of 0.02 mg/ml	[225]
Leucophyllum frutescens	Scrophulariaceae	Root, leaf	Methanol extracts against a drug-resistant strain of *M.* tuberculosis CIBIN/UMF15:99 with MIC of 62.5 *μ*g/ml	[202]
Maerua edulis	Capparaceae	Root	Hexane extracts against *M*. bovis BCG, *M*. tuberculosis H37Ra with MIC 31.2–62.5 *μ*g/ml	[226]
Mallotus philippensis	Euphorbiaceae	Leaf, fruit	Ethanolic extracts of fruit and leaves against *M*. tuberculosis H37Rv (ATCC 27294) both with MIC of 250 *μ*g/ml	[227]
Metrosideros excelsa	Myrtaceae	Leaf	Methanol extracts against *M*. smegmatis with IC_50_ of 0.11 mg/ml	[226]
Millettia stuhlmannii	Fabaceae	Leaf	Acetone extracts against *M*. smegmatis with MIC of 0.13 mg/ml	[228]
Morinda citrifolia	Rubiaceae	Leaf	Aqueous extract has an inhibition rate of 89% against *M.* tuberculosis H37Rv	[180]
Mucuna imbricata	Fabaceae	Seed	Methanol extracts have potential antimycobacterial activity and the synergistic group consisting of rifampicin in murine model	[185]
Murraya koenigii	Rutaceae	Leaf	Ethanol extracts against M. smegmatis with IC_50_ of 300 *μ*g/ml	[194]
Musa acuminata	Musaceae	Stem	Methanol extracts against drug-resistant variants of *M.* tuberculosis with MIC of 200 *μ*g/ml	[204]
Myoporum crassifolium	Scrophulariaceae	Wood	Hydrodistillation with essential oils against *M*. bovis BCG (strain 11-73 P2) with MIC of 50 *μ*g/ml	[186]
Myrica gale	Myricaceae	Root, stem	Ethyl acetate extracts with 96% inhibition at 100 *μ*g/ml against *M.* tuberculosis H37Rv (ATCC 27294)	[198]
Myristica fatua	Myricaceae	Almond	Dichloromethane soluble extracts against *M*. bovis BCG (strain 11-73 P2) with MIC of 50 *μ*g/ml	[186]
Nasturtium officinale	Cruciferae	Aerial parts	Chloroform extracts against two drug-resistant strains of *M.* tuberculosis with MIC of 50-100 *μ*M	[204]
Olea europaea	Oleaceae	Leaf	Hexane extracts against the drug-resistant variants of *M*. tuberculosis with MIC of 25-100 *μ*M	[204]
Otostegia integrifolia	Lamiaceae	Root	Chloroform extract of roots was the most active on *M.* tuberculosis H37Rv (MIC 156 *μ*g/ml) and AOZ8W-4 (MDR-TB clinical isolate) (MIC 0.078 mg/ml)	[229]
Pelargonium graveolens	Geraniaceae	Seed	Hydrodistillation for essential oil against tested isolates ranged from 19.5 *μ*g/ml to 78 *μ*g/ml	[230]
Pelargonium sidoides	Geraniaceae	Root	Aqueous extracts inhibiting the growth of *M*. tuberculosis H37Rv (ATCC 27294) by 96% at a sample concentration of 12.5 *μ*g/ml	[231]
Pentanisia prunelloides	Rubiaceae	Root	80% ethanol against *M*. tuberculosis H37Ra (ATCC 25177) with MICof 0.78 mg/ml	[215]
Persea americana	Lauraceae	Leaf, seed	Methanolic extracts against *M*. tuberculosis H37Ra with MIC of 31.2 *μ*g/ml and H37Rv; chloroformic extract of seeds against *M.* tuberculosis H37Rv MIC less than 50 *μ*g/ml	[229, 232]
Phymaspermum acerosum	Asteraceae	Root, leaf	Ethanol and water extracts had the best MIC value of 20 *μ*g/ml against five *M.* tuberculosis strains	[221]
Piper cernuum	Piperaceae	Leaf	Hydrodistillation with water displayed moderate activity against the *M*. tuberculosis H37Rv with MIC of 125 *μ*g/ml	[233]
Piper diospyrifolium	Piperaceae	Leaf	Hydrodistillation with water displayed moderate activity against the *M*. tuberculosis H37Rv with MIC of 125 *μ*g/ml	[233]
Piper guineense	Piperaceae	Seed	Methanol extracts against *M.* tuberculosis H37Ra and H37Rv with MIC of 256 *μ*g/ml	[210]
Piper imperiale	Piperaceae	Flower	Ethanolic extracts against *M*. tuberculosis H37Rv with MIC of 75 *μ*g/ml	[234]
Piper rivinoides	Piperaceae	Leaf	Hydrodistillation with water displayed moderate activity against the *M*. tuberculosis H37Rv with MIC of 125 *μ*g/ml	[233]
Piper sarmentosum	Piperaceae	Leaf	Extracts with petroleum ether, chloroform, and methanol, against *M.* tuberculosis H37Rv with MIC of 25, 25, and 12.5 *μ*g/ml	[235]
Pisonia borinquena	Nyctaginaceae	Leaf	95% ethanol extracts against *M*. tuberculosis H37Rv (ATCC 27294) with 85% inhibition at 100 *μ*g/ml	[216]
Pittosporum tenuifolium	Pittosporaceae	Leaf	Ethanol extracts against *M.* smegmatis with IC_50_ of 0.78 mg/ml	[225]
Pluchea indica	Asteraceae	Flower and leaf	80% methanol extract against *M*. tuberculosis H37Rv ATCC 25618 with an MIC of 800 *μ*g/ml	[191]
Plumbago zeylanica	Plumbaginaceae	Root	Ethanol extract against *M.* tuberculosis H37Rv (ATCC 27294) with MIC of 31.25 *μ*g/ml	[196]
Psychotria zombamontana	Rubiaceae	Leaf	Acetone extract decreased 256-fold of MIC in combination with fifampicin against *M*. aurum and reduction of the MICs of the anti-TB drug ranged from 2-fold to 4-fold, 2-fold to 64-fold, and 2-fold to 64-fold for *M*. smegmatis, *M.* aurium, and *M*. tuberculosis, respectively	[207]
Pterocarpus osun	Fabaceae (Leguminosae)	Stem	Chloroform extract against *M*. tuberculosis H37Rv and *M.* bovis BCG with MIC of 1225 *μ*g/ml and 1100 *μ*g/ml, respectively, by MABA	[67, 236]
Pterolobium stellatum	Fabaceae	Root	Chloroform extracts of roots were the most active on *M*. tuberculosis H37Rv (MIC 39 *μ*g/ml) and AOZ8W-4 (MDR-TB clinical isolate) (MIC 0.078 mg/ml)	[229]
Rhynchosia precatoria	Fabaceae	Root	Extracts by n-hexane, dichloromethane, ethyl acetate, and methanol individually against *M*. tuberculosis H37Rv with MIC of 15.6-125 *μ*g/ml	[212]
Ricinus communis	Euphorbiaceae	Seed	Hexane extracts against *M*. tuberculosis H37Rv sensitive strain with MIC of 2.5 mg/ml by MABA	[203]
Rosmarinus officinalis	Lamiaceae	Leaf	Ethanolic extracts against *M*. tuberculosis H37Ra with MIC of 6.25 *μ*g/ml	[224]
Satureja aintabensis	Lamiaceae	Aerial parts	Extraction with petroleum ether, ethyl acetate, and methanol killed *M*. tuberculosis with MIC of 50-800 *μ*g/ml	[237]
Satyrium nepalense	Orchidaceae	Flower	Hexane extracts against *M*. tuberculosis H37Rv TMC-102 with MIC of 15.7 *μ*g/ml	[238]
Schinus molle	Anacardiaceae	Fruit	Methanol extract against a drug-resistant strain of *M*. tuberculosis CIBIN/UMF15:99 with MIC of 125 *μ*g/ml	[202]
Securidaca longepedunculata	Polygalaceae	Root	Hexane extracts against *M*. bovis BCG, *M.* tuberculosis H37Ra, and H37Rv with 62.5 *μ*g/ml	[226]
Solanum torvum	Solanaceae	Leaf	80% ethanol crude extracts against *M*. tuberculosis H37Ra (ATCC 25177™) with MIC of 156.3 *μ*g/ml	[184]
Sphaeranthus indicus	Asteraceae	Floral head	Ethanol extract against *M*. tuberculosis H37Rv (ATCC 27294) with MIC of 31.25 *μ*g/ml	[196]
Sterculia setigera	Sterculiaceae	Leaf	Hexane, dichloromethane, and ethyl acetate extracts against *M.* tuberculosis H37Rv with MICs of 84 *μ*g/ml, 62 *μ*g/ml, and 128 *μ*g/ml, respectively	[239]
Swinglea glutinosa	Rutaceae	Fruit peel	Aqueous extracts for essential oils against *M*. tuberculosis H37Rv (ATCC 27294) with MIC of 100 *μ*g/ml	[182]
Tabernaemontana elegans	Apocynaceae	Root	Ethyl acetate extracts against *M*. tuberculosis H37Rv with MIC of 15.6 *μ*g/ml	[226]
Tabernaemontana coronaria	Apocynaceae	Leaf	Hexane partition from methanol extracts of MIC of 100 *μ*g/ml against *M.* tuberculosis H37Rv	[206]
Terminalia phanerophlebia	Combretaceae	Leaf, root, twig	80% ethanol against *M*. tuberculosis H37Ra (ATCC 25177) with 0.30 and 0.78 mg/ml, respectively	[215]
Terminalia sericea	Combretaceae	Stem bark	Acetone extracts against *M.* tuberculosis H37Ra with MIC of 25 *μ*g/ml	[197]
Thymus sibthorpii	Lamiaceae	Aerial parts	Extracts with petroleum ether, ethyl acetate, and methanol against *M*. tuberculosis with MIC of 50–800 *μ*g/ml	[237]
Trachyspermum copticum	Apiaceae	Aerial parts	Hydrodistillation extracts against *M*. kansasii and MDR-TB with MICs of 78 *μ*g/ml	[230]
Urtica dioica	Urticaceae	Leaf	Hexane extracts against *M*. smegmatis with MIC of 250 *μ*g/ml	[201]
Uvaria rufa	Annonaceae	Leaf	Lead acetate-treated crude chloroform extracts against *M*. tuberculosis H37Rv with MIC of 8 *μ*g/ml	[136]
Vetiveria zizanioides	Poaceae	Root	Ethanolic extract and hexane fraction 500 *μ*g/ml or 50 *μ*g/ml against *M*. tuberculosis H37Rv and H37Ra	[240]
Vismia baccifera	Clusiaceae	Leaf	Dichloromethane-methanol extracts (1.1) inhibit the *M*. tuberculosis H37Rv at 50 *μ*g/ml with 70.3 ± 0.5%	[188]
Xylopia aethiopica	Annonaceae	Fruit, bark	Methanol extracts against *M*. tuberculosis H37Ra and H37Rv with MIC of 512 *μ*g/ml	[210]
Zanthoxylum capense	Rutaceae	Root	Dichloromethane extracts against *M*. bovis BCG, *M.* tuberculosis H37Ra, and H37Rv with MICs of 31.2 *μ*g/ml	[226]
Zingiber officinale	Zingiberaceae	Rhizome	Ethanol extract against *M*. tuberculosis H37Ra with MIC of 2500 *μ*g/ml by MABA	[184]

^a^Minimum inhibitory concentration (MIC); ^b^microplate alamar blue assay (MABA); ^c^half maximal inhibitory concentration (C_50_).

**Table 3:** Important anti-TB traditional medicinal plants in literature by the systemic survey on the prescribed formula.

Species number	Family number	Main families	Country or region	References
13	10	Asteraceae (3), Chrysobalanaceae, Araliaceae, Acanthaceae, Chrysobalanaceae, Cucurbitaceae, Fabaceae, Lamiaceae, Melastomataceae, Phyllanthaceae, Polygonaceae	Burundian	[241]
9	8	Apocynaceae, Verbenaceae, Rubiaceae, Goodeniaceae, Agavaceae, Moraceae, Myrtaceae, Zingiberaceae	Manus Province, Papua New Guinea	[242]
184	77	Fabaceae (21), Asteraceae (12), Malvaceae (11)	Bapedi (South Africa)	[243]
30	21	Alliaceae (3), Rutaceaeare (3), Apiaceae (2), Caryophyllaceae (2), Asteraceae (2), Lamiaceae (2), Myrtaceae (2), Solanaceae (2)	Nkonkobe municipality, Eastern Cape Province (South Africa)	[244]
21	12	Asteraceae, Fabaceae, Geraniaceae	Mangaung metro, Thabo Mofutsanyana, and Lejweleputswa in South Africa	[245]
25	14	Fabaceae (5), Euphorbiaceae (3), Asteraceae (3), Lamiaceae (12%)	Bas-Congo Province, Democratic Republic of Congo	[208]
15	13	Amaryllidaceae (3), Xanthorrhoeaceae (2), Arecaceae (2), Solanaceae (2), Meliaceae, Acanthaceae, Poaceae, Phyllanthaceae, Melastomataceae, Poaceae, Cyperaceae, Zingiberaceae, Amaranthaceae, Asteraceae	Greater Accra and eastern communities in Ghana	[246]
95	48	Loranthaceae (6), Caesalpiniaceae (5), Papilionaceae (5), Poaceae (4), Mimosaceae (4), Scrophulariaceae (4), Anacardiaceae (3), Combretaceae (3), Liliaceae (3), and Solanaceae (3)	Niger state, Nigeria	[247]
66	35	Rutaceae (7), Euphorbiaceae (5), Rubiaceae (4), Anacardiaceae (3), Fabaceae (3), Verbenaceae (3), Arecaceae (2), Annonaceae (2), Solanaceae (2), Moraceae (2), Rhamnaceae (2)	Lao PDR	[248]
23	20	Arecaceae (2), Aristolochiaceae (2), Rubiaceae (2)	Malaysia	[249]
181	67	Asteraceae (31), Fabaceae (14), Lamiaceae (9), Euphorbiaceae (7), Celastraceae (5)	South Africa	[250]
62	38	Asteraceae (12), Aristolochiaceae (3), Compositae (3), Rosaceae (3), Juglandaceae (2), Zygophyllaceae (2), Verbenaceae (2), Rutaceae (2), Papaveraceae (2), Fabaceae (2)	Mexico	[10]
88	36	Lamiaceae (9), Asteraceae (7), Papilionaceae (4), Acanthaceae (3), Caesalpiniaceae (3), Capparaceae (3), Euphorbiaceae (3), Mimosaceae (3)	Districts of Kamuli, Kisoro, and Nakapiripirit in Uganda	[251]
90	44	Fabaceae (13), Asteraceae (7), Moraceae (5), Rutaceae (4)	Districts of Mpigi and Butambala, Uganda	[252]
35	22	Fabaceae (5), Rutaceae (4), Apocynaceae (3), Menispermaceae (3), Malvaceae (3)	Madhya Pradesh, India	[196]
132	45	Annonaceae (14), Zingiberaceae (12), Rutaceae (10), Annonaceae (10), Asteraceae (8), Euphorbiaceae (8), Fabaceae (7)	Southeast Asian	[253]
10	8	Fabaceae (3), Canellaceae (1), Rubiaceae (1), Anacardiaceae (1), Rutaceae (1), Myrtaceae (1), Merlucciidae (1), Guttiferae (1)	Lake Victoria Basin (Uganda, Kenya, and Tanzania)	[254]
14	8	Euphorbiaceae (4), Verbenaceae (3), Rutaceae (2)	Lake Victoria region and the Samburu community	[255]
2	2	Achillea millefolium (1), Dryopteris stewartii (1)	Kel village, Neelum Valley, Azad Kashmir, Pakistan	[256]
4	3	Amaryllidaceae (1), Lauraceae (1), Amaranthaceae (1), Asteraceae (1)	Sulaymaniyah Province, Kurdistan, Iraq	[257]
22	18	Liliaceae (3), Euphorbiaceae (2), Verbenaceae (2)	India	[258]
6	6	Vitaceae (1), Poaceae (1), Pinaceae (1), Musaceae (1), Rosaceae (1), Leguminosae (1)	Arabian Peninsula	[259]
2	2	Asteraceae (1), Dryopteridaceae (1)	Pakistan	[251]
70	44	Arecaceae (4), Euphorbiaceae (4), Fabaceae (3), Piperaceae (3), Rutaceae (3)	Malaysia	[191]
56	26	Ranunculaceae (8), Liliaceae (6), Asteraceae (5), Umbellifera (4), Campanulaceae (3), Rubiaceae (4), Rosaceae (2), Scrophulariaceae (2), Labiatae (2), Fabaceae (2)	China	[260]
82	30	Combretaceae (13), Fabaceae (12), Moraceae (6), Rubiaceae (5), Polygalaceae (5)	Nigeria	[261]

## Data Availability

All data included in this article are available from the corresponding author upon request.

## Abbreviations

TB: Tuberculosis
TM: Traditional medicines
WHO: World Health Organization
DTM: Department of Traditional Medicine
ICTM: International Classification of Traditional Medicine
ICD-11: International Classification of Diseases—11
TCM: Traditional Chinese Medicine
TAM: Traditional African medicine
XDR: Emergence of extensively drug-resistant
TDR: Total drug-resistant
MIC: Minimum inhibitory concentration
MBC: Minimum bactericidal concentration
MIA: Minimum inhibitory amount
IC_50_: Half maximal inhibitory concentration
MABA: Microplate alamar blue assay.
